# Burden of Hematological Malignancies in East Asia from 1990 to 2021

**DOI:** 10.3390/jcm14238381

**Published:** 2025-11-26

**Authors:** James Fan Wu, Frances Dominique V. Ho, Rod Carlo Columbres, Anthony Tudisco, Urvish Jain, Aryan Selokar, Nishwant Swami, Bhav Jain, Ji Hyun Hong, Erin Jay G. Feliciano, Frederic Ivan L. Ting, Edward Christopher Dee

**Affiliations:** 1 Division of Hematology and Oncology, Department of Medicine, Medical College of Wisconsin, 8701 Watertown Plank Rd, Milwaukee, WI 53226, USA; 2 College of Medicine, University of the Philippines, Manila 1000, Philippines; fvho@up.edu.ph; 3 Health Economics and Finance Program, Philippine Institute for Development Studies, Quezon City 1101, Philippines; 4 Genitourinary Malignancies Branch, Center for Cancer Research, National Cancer Institute, National Institutes of Health, Bethesda, MD 20892, USA; rodcarlo.columbres@gmail.com; 5 Department of Medicine, Alta Bates Summit Medical Center Sutter Health, Oakland, CA 94609, USA; 6 Department of Computational & Systems Biology, University of Pittsburgh, Pittsburgh, PA 15260, USA; awt28@pitt.edu (A.T.); urj2@pitt.edu (U.J.); 7 University of California San Diego, La Jolla, CA 92093, USA; aryanselokar@gmail.com; 8 Division of Internal Medicine, Hospital of the University of Pennsylvania, Philadelphia, PA 19104, USA; nishwant.swami@pennmedicine.upenn.edu; 9 Stanford School of Medicine, Stanford, CA 94305, USA; bhavjain@stanford.edu; 10 Department of Radiation Oncology, College of Medicine, The Catholic University of Korea, Seoul 06591, Republic of Korea; rojihyunhong@gmail.com; 11 School of Medicine and Public Health, Ateneo de Manila University, Pasig City 1604, Philippines; feliciae2@nychhc.org; 12 Department of Medicine, NYC Health + Hospitals/Elmhurst, Icahn School of Medicine at Mt. Sinai, Queens, NY 11373, USA; 13 Division of Oncology, Department of Medicine, Corazon Locsin Montelibano Memorial Regional Hospital, Bacolod 6100, Philippines; f.ting@usls.edu.ph; 14 Department of Clinical Sciences, College of Medicine, University of St. La Salle, Bacolod 6100, Philippines; 15 Department of Radiation Oncology, Memorial Sloan Kettering Cancer Center, New York, NY 10065, USA; deee1@mskcc.org

**Keywords:** global burden of disease, epidemiology, hematological malignancies, Asia

## Abstract

**Introduction:** Hematological malignancies (HMs) represent a diverse spectrum of hematopoietic and lymphoid neoplasms involving the blood, bone marrow, and lymphatic organs. Better understanding of the burden of HMs in East Asia will allow for more targeted policy and public health efforts. **Methods:** Using the Global Burden of Diseases, Injuries, and Risk Factors Study 2021, we extracted 2021 and 1990–2021 trend estimates for total counts and age-standardized rates (per 100,000 person-years) for incidence, mortality, and disability-adjusted life years (DALYs) for leukemia and subtypes, multiple myeloma (MM), non-Hodgkin lymphoma (NHL), and Hodgkin lymphoma (HL) in East Asia (China, Japan, North Korea, Republic of Korea, Mongolia, and Taiwan). **Results:** In 2021, the burden of HMs globally and in East Asia was driven by China and Japan. China had the highest global HM burden with 238,051 new cases, 117,188 deaths, and 3.9 million DALYs. NHL and leukemia accounted for the majority of new cases, deaths, and DALYs in all countries. From 1990 to 2021, in most HMs and countries the age-standardized incidence rate (ASIR) increased, while the age-standardized mortality rate (ASMR) and age-standardized DALY rate (ASDALYR) decreased. The notable exception is the significant increase of MM ASIR, ASMR, and ASDALYR in Mongolia, Taiwan, and China, with increases in China by 200–300%. **Conclusions:** With the significant contribution to the global burden of HMs from China and Japan, diagnosis and treatment of HMs in these two countries should be a primary global health focus. The significant relative increase of MM ASIR, ASMR, and DALYs across many East Asia countries, especially in China, highlights MM as an important public health focus. Significant variations between the other East Asia countries also warrant further country- and disease-specific investigations.

## 1. Introduction

With global shifts in lifestyle, dietary, and environmental exposures, coupled with improvements in access to health care, health systems worldwide currently grapple with a rise in the burden of cancer [1,2], especially hematological malignancies (HMs) [3,4,5]. HMs represent a diverse spectrum of hematopoietic and lymphoid neoplasms involving the blood, bone marrow, and lymphatic organs [6]. The major types of HMs are leukemia, multiple myeloma (MM), non-Hodgkin lymphoma (NHL), and Hodgkin lymphoma (HL), with each one exhibiting distinct clinical, histopathologic, and molecular profiles [6].

Previous cancer registry studies have shown that while the incident cases of HMs have increased since 1990, reaching 1.3 million in 2019, the mortality rate has generally declined, which may reflect advancements in their prevention, timely diagnosis, and management [5,7,8]. However, such research, which described and compared the global HM epidemiology and temporal trends, broadly focused on world regions and territories. There remains a need to understand within-region differences in HM disease burden to facilitate more targeted policy and public health efforts.

Home to over 1.6 billion people, East Asia is a vast region composed of China, Japan, Mongolia, North Korea, Republic of Korea, and Taiwan [9]. While these neighboring countries may share some similarities in genetic backgrounds and culture, they remain incredibly diverse, particularly in terms of socio-economic development and health systems. In terms of cancer, East Asia contributed the highest number of incident cancer cases (6,008,355 per year) and deaths (3,617,104 per year) compared to other Asian regions in 2020 [10]. Despite this, epidemiologic studies of HMs in the region have been limited, with local studies primarily describing individual HMs [11,12,13,14,15,16,17]. In this study, we utilize estimates from the Global Burden of Diseases, Injuries, and Risk Factors Study 2021 (GBD 2021) to shed light on the epidemiologic patterns of HMs in East Asia and its constituent countries, with the hope of informing disease prevention and control strategies in the region.

## 2. Methods

### 2.1. Data Source

The Global Burden of Diseases, Injuries, and Risk Factors Study 2021 (GBD 2021) contains statistical data for 371 diseases and injuries and 204 countries and territories [18,19,20]. The data sources and methods used in generating cancer estimates have been described in previously published GBD 2021 studies [18]. The data are available online from the Global Health Data Exchange (GHDx) query tool [https://vizhub.healthdata.org/gbd-results/ (accessed on 20 August 2024)]. Data sources and code are available online (https://ghdx.healthdata.org/gbd-2021/sources and https://ghdx.healthdata.org/gbd-2021/code). We extracted cancer estimates from the GBD 2021 study on HMs in East Asia (China, Japan, North Korea, Republic of Korea, Mongolia, and Taiwan) as classified by the United Nations Statistical Division [9]. Of note, Taiwan is not shown separately from China in the list of nations by the United Nations. GBD produces estimates for Taiwan and China; therefore, Taiwan is categorized in the East Asia region. Data on leukemia, MM, NHL, and HL were extracted for China, Japan, North Korea, Republic of Korea, Mongolia, and Taiwan. GBD also presents data on leukemia subtypes; thus, data on acute myeloid leukemia (AML), acute lymphoid leukemia (ALL), chronic myeloid leukemia (CML), chronic lymphoid leukemia (CLL), and other leukemia were also extracted. For each disease and location, the total number and age-standardized rates per 100,000 person-years of incidence cases, deaths, and disability-adjusted life years (DALY) by sex and age from 1990 to 2021 with relative percentage changes over time were extracted.

### 2.2. Statistical Analysis and Data Visualization

To increase the comparability between countries, age-standardization was used, which accounts for differences in population age structures. DALYs were calculated by adding years of life lost due to premature mortality and years lived with disability, with one DALY representing the loss of one year of full health. All the estimates are means reported with 95 percent uncertainty intervals (UIs) inside square brackets. GBD calculates UIs based on 2.5th and 97.5th percentiles from 1000 draws in each modelling step. Relative percentage changes from 1990 to 2021 were also reported with statistical significance determined when the 95 percent UI did not cross zero. Male-to-female ratios from 1990 to 2021 for ASRs were calculated using Microsoft Excel to assess the change of HM burden by sex over time. Maps of East Asia for all age-standardized rates for each HM were generated using the GBD Compare visualization tool [https://vizhub.healthdata.org/gbd-compare/ (accessed on 20 August 2024)]. Line graphs generated using the Global Health Data Exchange (GHDx) query tool [https://vizhub.healthdata.org/gbd-results/ (accessed on 20 August 2024)]. and Microsoft Excel were used to show changing trends of incidence, death, DALYs, and sex burden over time. Histograms generated using GraphPad Prism (version 8) were used to show differences of burden incidence, death, and DALYs between 1990 and 2021 by age (5-year age intervals).

## 3. Results

### 3.1. East Asia

**Figure 1:**
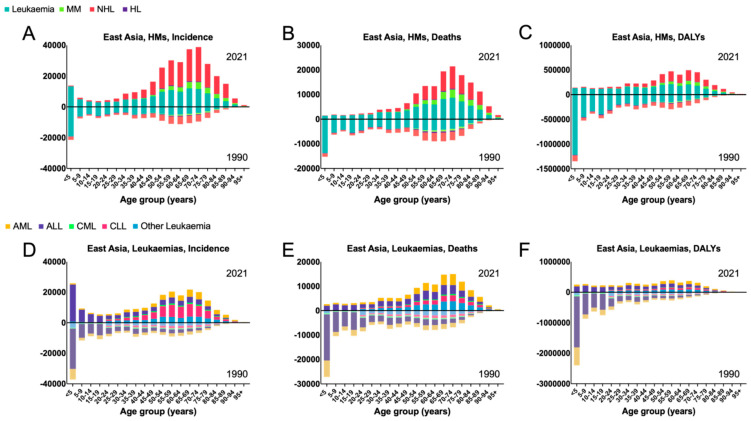
East Asia—hematological malignancy burden by age, 1990 and 2021. (**A**) Incident cases, (**B**) Deaths, and (**C**) DALYs for leukemia, MM, NHL, and HL. (**D**) Incident cases, (**E**) Deaths, and (**F**) DALYs for AML, ALL, CML, CLL, and other leukemia.

For East Asia, the trends from 1990 to 2021 for incident cases, deaths, and DALYs in hematological malignancies are presented in Table 1. In 2021, for East Asia cumulatively and for each individual country, leukemia and NHL were responsible for the most incident cases, deaths, and DALYs. From 1990 to 2021, MM and NHL both demonstrated relative increases across all measures; leukemia demonstrated a relative increase in incident cases but decreases in deaths and DALYs; and HL demonstrated relative decreases across all measures. MM and NHL demonstrated the largest and second-largest relative increases of 375.2% and 291.3% in incident cases, 330.7% and 97.0% in deaths, and 291.3% and 39.4% in DALYs, respectively. HL demonstrated the largest relative decreases across all measures: −2.2% in incident cases, −39.8% in deaths, and −56.4% in DALYs (Table 1). Among all age groups in 2021 (Figure 1A–C), the most incident cases occurred in the <5 group for leukemia, the 70–74 group for MM and NHL, and the 65–69 group for HL. The most deaths occurred in the 55–59 group for leukemia, the 70–74 group for MM and NHL, and the 65–69 group for HL. The most DALYs occurred in the 55–59 group for leukemia and HL compared to the 65–69 group for MM and NHL.

For East Asia, the trends from 1990 to 2021 for incident cases, deaths, and DALYs in leukemia subtypes are presented in Supplementary Table S1. In 2021, ALL accounted for the most incident cases and DALYs, whereas AML accounted for the most deaths. From 1990 to 2021, all leukemia subtypes demonstrated relative increases in incident cases except for CML; relative increases in deaths except for ALL and CML; and relatives decreases in DALYs except for CLL. CLL demonstrated the largest relative increases across all measures: 314.5% in incident cases, 80.3% in deaths, and 52.8% in DALYs. Conversely, CML demonstrated the largest relative decreases across all measures: −13.8% in incident cases, −46.5% in deaths, and −65.9% in DALYs. Among all age groups in 2021 (Figure 1D–F), the most incident cases occurred in the 70–74 group for AML, the <5 group for ALL, the 55–59 group for CML, the 65–69 group for CLL, and the 50–54 group for other leukemia. The most deaths occurred in the 65–69 group for AML, ALL, and other leukemia compared to the 70–74 group for CML and CLL. The most DALYs occurred in the 55–59 group for AML and CML, the 5–9 group for ALL, and the 65–69 group for CLL and other leukemia.

Maps of age-standardized rates of incident cases (ASIR), deaths (ASMR), and DALYs (ASDALY) in East Asia in 2021 are presented in Supplementary Figure S1 for leukemia, MM, NHL, and HL, and Supplementary Figure S2 for leukemia subtypes. In terms of sex, the incidence, deaths, and DALYs are higher in males than in females in all four HMs and five leukemia subtypes (Supplementary Figures S3 and S4).

### 3.2. China

In 2021, China was the leading contributor to the global burden of hematological malignancies, with 238,051 new cases, 117,188 deaths, and 3.9 million DALYs. Globally, China demonstrated the most incident cases, deaths, and DALYs for leukemia and NHL. For MM globally, China ranked second in incident cases and deaths after the United States and first in DALYs. For HL globally, China ranked third in incident cases, second in deaths, and fourth in DALYs.

**Figure 2:**
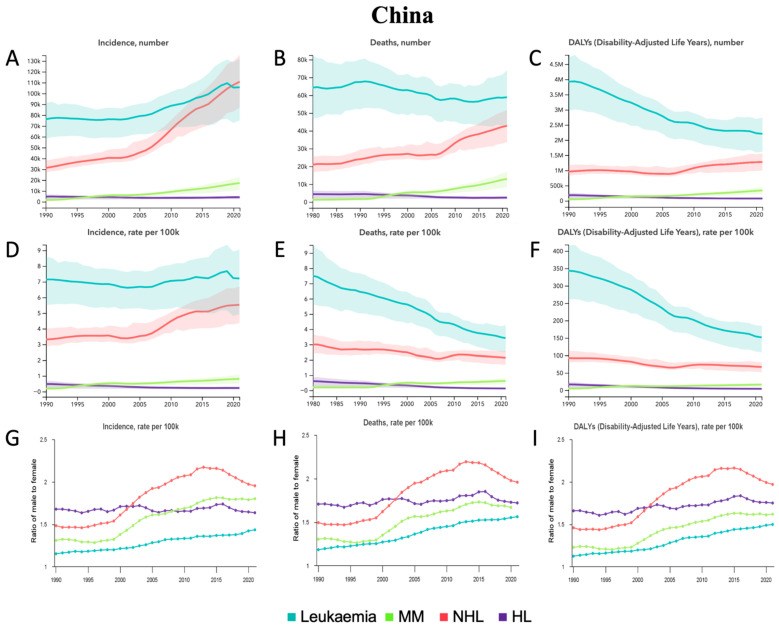
China—trends in hematological malignancy burden from 1990 to 2021. (**A**) Incident cases, (**B**) Deaths, and (**C**) DALYs for leukemia, MM, NHL, and HL. (**D**) Age-standardized incidence rate (ASIR), (**E**) Age-standardized mortality rate (ASMR), and (**F**) Age-standardized DALY rate (ASDALY) for leukemia, MM, NHL, and HL. (**G**) Ratio of male-to-female ASIR, (**H**) Ratio of male-to-female ASMR, and (**I**) Ratio of male-to-female ASDALYR for leukemia, MM, NHL, and HL.

From 1990 to 2021, incident cases increased across all HMs except for HL, with the largest relative percent increases in MM of 918.7% [310.4–1657.5] and in NHL of 255.3% [172.7–367.9] (Table 1, Figure 2A). Deaths demonstrated a relative percent increase in MM of 716.0% [229.5–1314.1] and NHL of 78.4% [37.2–134.7] (Table 1, Figure 2B). DALYs demonstrated a relative percent increase in MM and NHL but a decrease in leukemia and HL, with the most significant change being a 622.1% [192.7–1140.6] relative percent increase in MM (Table 1, Figure 2C). From 1990 to 2021, ASIR demonstrated a relative percent increase in MM of 312.5% [62.9–620.9] and in NHL of 66.7% [28.7–117.0] but a decrease in HL of −52.5% [−67.8 to −13.6] (Table 1, Figure 2D). ASMR demonstrated a relative percent increase in MM of 221.5% [26.3–463.3] but a decrease in leukemia of −47.0% [−56.7 to −31.4] and in HL of −73.2% [−81.8 to −50.3] (Table 1, Figure 2E). ASDALYR demonstrated a relative percent increase in MM of 221.1% [28.5–459.2] but a decrease in leukemia of −55.9% [−65.3 to −41.6], in NHL of −27.6% [−44.5 to −5.7], and in HL of −75.4% [−83.5 to −54.6] (Table 1, Figure 2F). In terms of sex from 1990 to 2021, the male-to-female ratio for ASRs in leukemia, MM, and NHL increased across all measures (Figure 2G–I). Among all age groups in 2021 (Supplementary Figure S5A–C), the most incident cases occurred in the <5 group for leukemia compared to the 65–69 group for MM, NHL, and HL. The most deaths occurred in the 65–69 group for leukemia compared to the 70–74 group for MM, NHL, and HL. The most DALYs occurred in the 55–59 group for leukemia, NHL, and HL compared to the 65–69 group for MM.

From 1990 to 2021, CLL demonstrated relative percent increases in incident cases, deaths, and DALYs, with the largest increase in incident cases of 326.6% [215.2–503.5] (Supplementary Table S1, Supplementary Figure S6A–C). ALL and CML demonstrated relative percent decreases in deaths and DALYs (Supplementary Table S1, Supplementary Figure S6A–C). For age-standardized rates, AML demonstrated a relative percent decrease in ASDALYR; ALL demonstrated a decrease for ASMR and ASDALYR; CML demonstrated a decrease in ASIR, ASMR, and ASDALYR; CLL demonstrated an increase in ASIR; and other leukemia demonstrated a decrease in ASMR and ASDALYR (Supplementary Table S1, Supplementary Figure S6D–F). In terms of sex from 1990 to 2021, the male-to-female ratio for all leukemia sub-types increased (Supplementary Figure S6G–I). Among all age groups in 2021 (Supplementary Figure S5D–F), the most incident cases occurred in the 65–69 group for AML and CLL, the <5 group for ALL, the 55–59 group for CML, and the 50–54 group for other leukemia. The most deaths occurred in the 65–69 group for AML, ALL, and other leukemia compared to the 70–74 group for CML and CLL. The most DALYs occurred in the 55–59 group for AML and CML, the 65–69 group for CLL and other leukemia, and the 5–9 group for ALL.

### 3.3. Japan

In 2021, Japan was also a significant contributor to the global burden of hematological malignancies, with the fourth most new cases of 56,702 and deaths of 31,907, while having the seventh most DALYs of 571,070. For leukemia globally, Japan ranked fifth in incident cases and deaths, and tenth in DALYs. For MM globally, Japan ranked fifth in incident cases, fourth in deaths, and sixth in DALYs. For NHL globally, Japan ranked fourth in incident cases and deaths, and fifth in DALYs. For HL globally, Japan ranked 27th in incident cases, 22nd in deaths, and 40th in DALYs.

**Figure 3:**
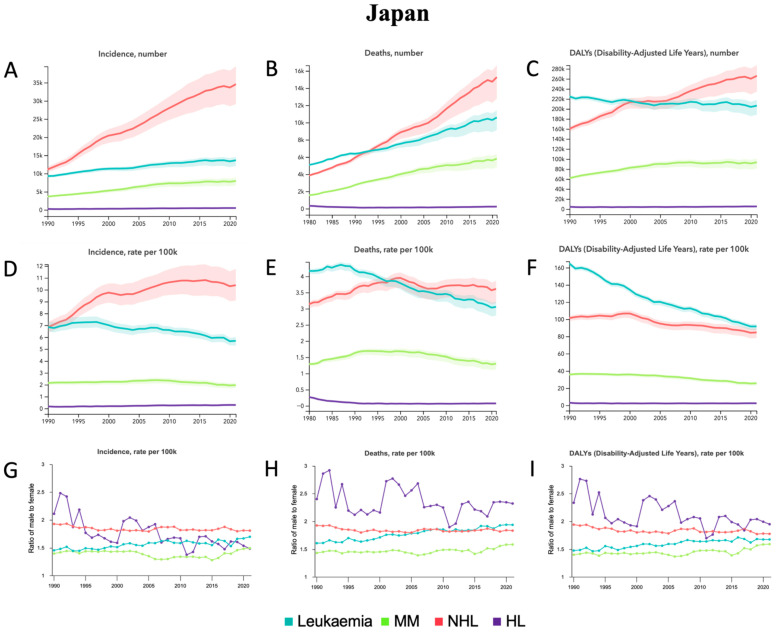
Japan—trends in hematological malignancy burden from 1990 to 2021. (**A**) Incident cases, (**B**) Deaths, and (**C**) DALYs for leukemia, MM, NHL, and HL. (**D**) Age-standardized incidence rate (ASIR), (**E**) Age-standardized mortality rate (ASMR), and (**F**) Age-standardized DALY rate (ASDALY) for leukemia, MM, NHL, and HL. (**G**) Ratio of male-to-female ASIR, (**H**) Ratio of male-to-female ASMR, and (**I**) Ratio of male-to-female ASDALYR for leukemia, MM, NHL, and HL.

From 1990 to 2021, incident cases, deaths, and DALYs increased across all HMs, except for a slight relative decrease in DALYs in leukemia of −8.1% [−15.1 to −3.8] (Table 1). The largest relative percentage increases were in MM and NHL across all measures: 118.1% [92.5–141.8] and 210.5% [165.7–261.9] for incident cases (Table 1, Figure 3A), 111.2% [87.1–125.7] and 157.9% [127.1–174.4] for deaths (Table 1, Figure 3B), and 51.0% [37.0–59.5] and 66.0% [50.3–75.4] for DALYs (Table 1, Figure 3B), respectively. From 1990 to 2021, across all age-standardized rates, the only relative percentage increases were in ASIR of 51.9% [33.7–74.1] in NHL (Table 1, Figure 3D) and 69.4% [55.3–84.4] in HL (Table 1, Figure 3D). With the exception of ASIR in MM and ASMR in NHL, which demonstrated no change, all other ASRs demonstrated relative percentage decreases from 1990 to 2021 (Table 1). Leukemia demonstrated the largest relative decreases across all ASRs, with decreases of −16.9% [−23.5 to −10.9] for ASIR (Table 1, Figure 3D), −27.1% [−31.1 to −24.5] for ASMR (Table 1, Figure 3E), and −44.4% [−46.3 to −42.8] for ASDALYR (Table 1, Figure 3F). In terms of sex from 1990 to 2021, the male-to-female ratio for ASRs increased for leukemia and MM, decreased for NHL, and was variable for HL (Figure 3G–I). Among all age groups in 2021 (Supplementary Figure S7A–C), most incident cases occurred in the 70–74 group for leukemia, NHL, and HL compared to the 80–84 group for MM. The most deaths occurred in the 80–84 group for leukemia, NHL, and HL compared to the 85–89 group for MM. The most DALYs occurred in the 70–74 for all HMs.

From 1990 to 2021, AML, CLL, and other leukemias demonstrated relative percentage increases for incident cases, deaths, and DALYs, except for DALYs in AML, which did not have any significant change (Supplementary Table S1, Supplementary Figure S8A–C). The largest relative increases were in CLL and other leukemia: 134.4% [111.8–154.2] and 217.5% [188.8–243.8] for incident cases, 117.1% [91.2–134.1] and 244.3% [211.4–267.1] for deaths, and 62.5% [46.1–74.1] and 125.2% [107.1–137.6] for DALYs, respectively (Supplementary Table S1, Supplementary Figure S8A–C). ALL and CML demonstrated relative percentage decreases for incident cases, deaths, and DALYs, with CML demonstrating the largest relative decreases of −44.2% [−50.9 to −37.9], −45.1% [−53.5 to −40.0], and −72.3% [−75.3 to −70.1], respectively (Supplementary Table S1, Supplementary Figure S8A–C). For age-standardized rates, AML, ALL, CML, and CLL demonstrated relative percentage decreases in ASIR, ASMR, and ASDALYR, with the exception of ASIR in ALL, which did not show any significant change, and ASIR in CLL, which showed an increase of 8.0% [0.6–15.9] (Supplementary Table S1, Supplementary Figure S8D–F). CLL demonstrated the largest relative percentage decreases. Other leukemia demonstrated relative percentage increases in all ASRs. In terms of sex from 1990 to 2021, the male-to-female ratio for ASRs increased for AML, ALL, CML, CLL, and other leukemias, except for ASIR for CML, which was stable over time (Supplementary Figure S8G–I). Among all age groups in 2021 (Supplementary Figure S7D–F), the most incident cases occurred in the 80–84 group in AML, the 70–74 group in ALL and other leukemia, the 85–89 group in CML, and the 75–79 group in CLL. The most deaths occurred in the 80–84 group in AML, the 70–74 group in ALL and other leukemia, and the 85–89 group in CML and CLL. The most DALYs occurred in the 70–74 group in AML, ALL, and other leukemias compared to the 85–89 group in CML and the 75–79 group in CLL.

### 3.4. North Korea

**Figure 4:**
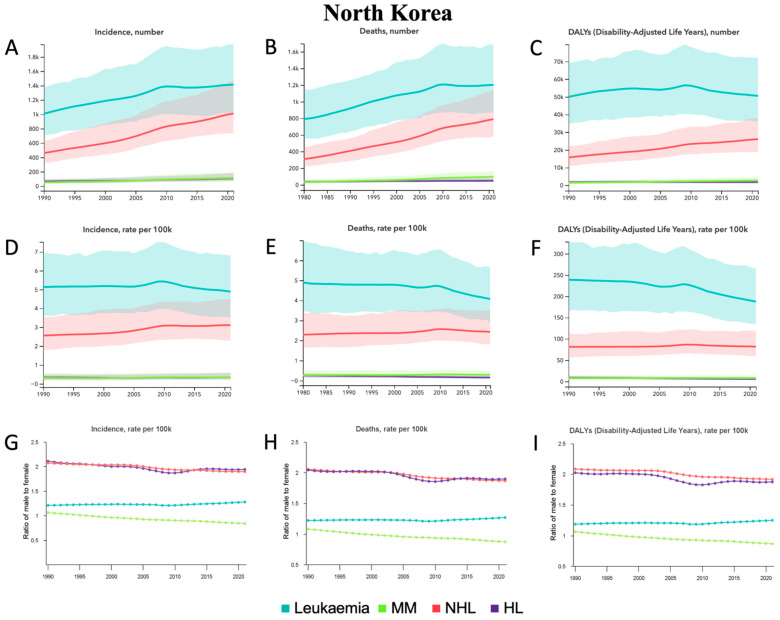
North Korea—trends in hematological malignancy burden from 1990 to 2021. (**A**) Incident cases, (**B**) Deaths, and (**C**) DALYs for leukemia, MM, NHL, and HL. (**D**) Age-standardized incidence rate (ASIR), (**E**) Age-standardized mortality rate (ASMR), and (**F**) Age-standardized DALY rate (ASDALY) for leukemia, MM, NHL, and HL. (**G**) Ratio of male-to-female ASIR, (**H**) Ratio of male-to-female ASMR, and (**I**) Ratio of male-to-female ASDALYR for leukemia, MM, NHL, and HL.

From 1990 to 2021, only MM and NHL demonstrated significant changes in incident cases, deaths, and DALYs, with relative percentage increases in MM of 124.5% [42.2–239.1], 112.4% [33.3–218.7], and 96.8% [24.7–196.0], respectively, and in NHL of 119.6% [55.0–210.8], 93.5% [34.8–171.9], and 65.6% [14.5–140.0], respectively (Table 1, Figure 4A–C). There were no significant changes in ASRs for any HM (Table 1, Figure 4D–F). In terms of sex from 1990 to 2021, the male-to-female ratio for ASIR, ASMR, and ASDALYR increased in leukemia but decreased in MM, NHL, and HL (Figure 4G–I). Among all age groups in 2021 (Supplementary Figure S9A–C), the most incident cases occurred in the 50–54 group for leukemia and HL compared to the 60–64 group for MM and the 55–59 group for NHL. The most deaths occurred in the 55–59 group for leukemia and NHL compared to the 60–64 group for MM and HL. The most DALYs occurred in the 50–54 group for leukemia, NHL, and HL compared to the 60–64 group for MM.

From 1990 to 2021, CLL demonstrated the largest relative percentage increases in incident cases of 165.3% [55.6–314.8], deaths of 102.6% [23.4–212.4], and DALYs of 88.3% [5.7–207.3] (Supplementary Table S1, Supplementary Figure S10A–C). Other leukemias also demonstrated relative percentage increases in incident cases of 72.6% [12.8–162.8] and deaths of 69.4% [13.6–160.1] (Supplementary Table S1, Supplementary Figure S10A–C). There were no other significant changes over time for total counts. There were also no significant changes in ASRs for any HM (Supplementary Table S1, Supplementary Figure S10D–F). In terms of sex from 1990 to 2021, the male-to-female ratio for ASIR, ASMR, and ASDALYR decreased for AML and CML, increased for ALL, and was stable for CLL (Supplementary Figure S10G–I). In terms of sex from 1990 to 2021, the male-to-female ratio for other leukemia was stable for ASIR but decreased for ASMR and ASDALYR. Among all age groups in 2021 (Supplementary Figure S9D–F), the most incident cases occurred in the 50–54 group for AML, CML, and other leukemia compared to the 55–59 group in ALL and the 60–64 group in CLL. The most deaths occurred in the 60–64 group for AML and CLL, the 55–59 group for ALL and CML, and the 50–54 group for other leukemia.

### 3.5. Republic of Korea

**Figure 5:**
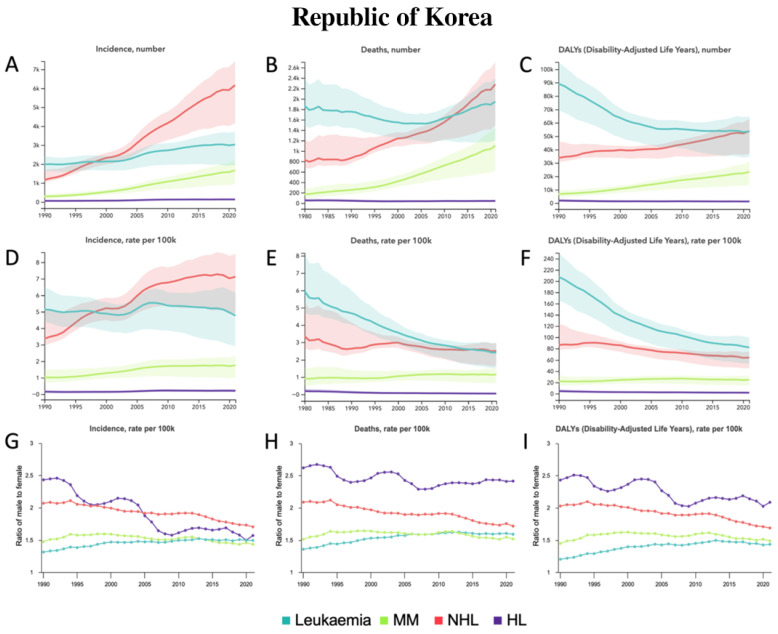
Republic of Korea—trends in hematological malignancy burden from 1990 to 2021. (**A**) Incident cases, (**B**) Deaths, and (**C**) DALYs for leukemia, MM, NHL, and HL. (**D**) Age-standardized incidence rate (ASIR), (**E**) Age-standardized mortality rate (ASMR), and (**F**) Age-standardized DALY rate (ASDALY) for leukemia, MM, NHL, and HL. (**G**) Ratio of male-to-female ASIR, (**H**) Ratio of male-to-female ASMR, and (**I**) Ratio of male-to-female ASDALYR for leukemia, MM, NHL, and HL.

From 1990 to 2021, incident cases increased for all HMs, with large relative percentage increases of 487.7% [187.5–758.6] in MM, 426.0% [173.2–583.9] in NHL, and 125.2% [30.0–242.5] in HL (Table 1, Figure 5A). Only MM and NHL demonstrated changes in deaths, with large relative percentage increases of 334.2% [111.9–539.5] and 163.8% [27.1–233.0], respectively (Table 1, Figure 5B). MM also demonstrated a large relative percentage increase of 247.4% [82.6–413.5] in DALYs compared to relative percentage decreases of −40.0% [−57.6 to −19.7] in leukemia and −34.2% [−62.1 to −1.3] in HL (Table 1, Figure 5C). From 1990 to 2021, for all ASRs, only NHL demonstrated a relative percentage increase in ASIR of 111.7% [2.6–173.9] (Table 1, Figure 5D). All other relative percentage changes in ASRs were decreases, including decreases in ASMR for leukemia and HL, and in ASDALYR for leukemia, NHL, and HL (Table 1, Figure 5E,F). In terms of sex from 1990 to 2021, the male-to-female ratio for ASIR, ASMR, and ASDALYR increased for leukemia, decreased for NHL and HL, and did not show any significant trends for MM (Figure 5G–I). Among all age groups in 2021 (Supplementary Figure S11A–C), the most incident cases occurred in the 75–79 group for leukemia and MM, the 60–64 group for NHL, and the 50–54 group for HL. The most deaths occurred in the 75–79 group for leukemia, MM, and HL compared to the 80–84 group for NHL. The most DALYs occurred in the 60–64 group for leukemia, NHL, and HL compared to the 65–69 group for MM.

From 1990 to 2021, CLL demonstrated relative percentage increases in incident cases of 354.5% [161.4–670.9] and deaths of 95.4% [16.4–211.8] (Supplementary Table S1, Supplementary Figure S12A,B). ALL and CML demonstrated relative percentage decreases in deaths and DALYs (Supplementary Table S1, Supplementary Figure S12B,C). For ASRs, CML and other leukemia demonstrated relative percentage decreases in ASIR (Supplementary Table S1, Supplementary Figure S12D). All leukemia subtypes demonstrated relative percentage decreases in ASMR and ASDALYR, except for the ASMR in AML, with the largest relative decreases in ALL and CML (Supplementary Table S1, Supplementary Figure S12E,F). In terms of sex from 1990 to 2021, the male-to-female ratio for ASIR, ASMR, and ASDALYR increased for all leukemia subtypes except CML, which did not have any clear trends (Supplementary Figure S12G–I). Among all age groups in 2021 (Supplementary Figure S11D–F), the most incident cases occurred in the 75–79 group for AML and CLL, the <5 group for ALL, the 50–54 group for CML, and the 60–64 group for other leukemia. The most deaths occurred in the 75–79 group for AML, the 60–64 group for ALL, and the 80–84 group for CML, CLL, and other leukemia. The most DALYs occurred in the 60–64 group for AML and other leukemia, the 20–24 group for ALL, the 50–54 group for CML, and the 75–79 group for CLL.

### 3.6. Mongolia

**Figure 6:**
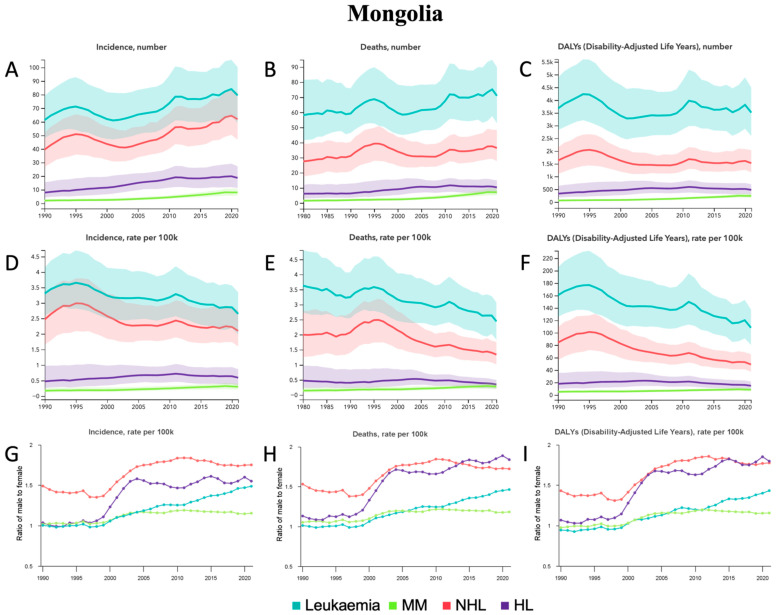
Mongolia—trends in hematological malignancy burden from 1990 to 2021. (**A**) Incident cases, (**B**) Deaths, and (**C**) DALYs for leukemia, MM, NHL, and HL. (**D**) Age-standardized incidence rate (ASIR), (**E**) Age-standardized mortality rate (ASMR), and (**F**) Age-standardized DALY rate (ASDALY) for leukemia, MM, NHL, and HL. (**G**) Ratio of male-to-female ASIR, (**H**) Ratio of male-to-female ASMR, and (**I**) Ratio of male-to-female ASDALYR for leukemia, MM, NHL, and HL.

From 1990 to 2021, MM demonstrated significant relative percentage increases of 312.9% [182.7–547.0] in incident cases, 279.0% [165.2–490.4] in deaths, and 295.7% [172.3–523.7] in DALYs (Table 1, Figure 6A–C). NHL and HL also demonstrated increases in incident cases (Table 1, Figure 6A). MM also demonstrated significant relative percentage increases in ASRs of 77.1% [23.6–173.9] in ASIR, 66.5% [18.0–157.6] in ASMR, and 68.9% [17.6–164.4] in ASDALYR (Table 1, Figure 6D–F). ASMR and ASDALYR decreased in NHL, and ASDALYR decreased in leukemia (Table 1, Figure 6E,F). In terms of sex from 1990 to 2021, the male-to-female ratio for ASIR, ASMR, and ASDALYR increased for all HMs (Figure 6G–I). Among all age groups in 2021 (Supplementary Figure S13A–C), the most incident cases occurred in the 55–59 group for leukemia and NHL, the 60–64 group for MM, and the 35–39 group for HL. The most deaths occurred in the 55–59 group for leukemia, NHL, and HL compared to the 60–64 group for MM. The most DALYs occurred in 10–14 group for leukemia, the 55–59 group for MM and NHL, and the 30–34 group for HL.

From 1990 to 2021, Mongolia demonstrated few changes for leukemia subtypes (Supplementary Table S1, Supplementary Figure S14A–F). AML and CLL demonstrated relative percentage increases in incident cases. ALL demonstrated a relative percentage decrease in DALYs and in all ASRs. In terms of sex from 1990 to 2021, the male-to-female ratio for ASIR, ASMR, and ASDALYR increased for AML, ALL, CML, and other leukemia (Supplementary Figure S14G–I). For CLL, the ASIR and ASMR decreased, while the ASDALYR increased. Among all age groups in 2021 (Supplementary Figure S13D–F), the most incident cases occurred in 55–59 group for AML, CLL, and other leukemia compared to the <5 group for ALL and the 45–49 group for CML. The most deaths occurred in the 55–59 group for AML, CML, and other leukemia compared to the 10–14 group for ALL and the 60–64 group for CLL. The most DALYs occurred in the 10–14 group for AML and ALL, the 45–49 group for CML, the 55–59 group for CLL, and the 50–54 group for other leukemia.

### 3.7. Taiwan

**Figure 7:**
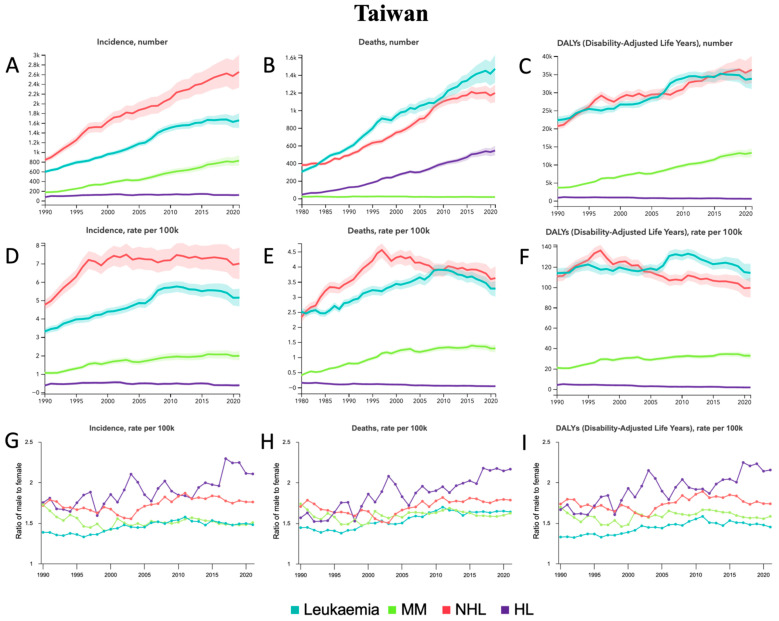
Taiwan—trends in hematological malignancy burden from 1990 to 2021. (**A**) Incident cases, (**B**) Deaths, and (**C**) DALYs for leukemia, MM, NHL, and HL. (**D**) Age-standardized incidence rate (ASIR), (**E**) Age-standardized mortality rate (ASMR), and (**F**) Age-standardized DALY rate (ASDALY) for leukemia, MM, NHL, and HL. (**G**) Ratio of male-to-female ASIR, (**H**) Ratio of male-to-female ASMR, and (**I**) Ratio of male-to-female ASDALYR for leukemia, MM, NHL, and HL.

From 1990 to 2021, leukemia, MM, and NHL all demonstrated relative percentage increases in incident cases, deaths, and DALYs (Table 1, Figure 7A–C). MM demonstrated the largest relative increases of 374.5% [304.7–439.6] in incident cases, 329.1% [278.7–380.1] in deaths, and 266.9% [225.9–307.2] in DALYs. While DALYs increased in leukemia and NHL, incident cases and deaths, like MM, also increased significantly by 178.3% [148.6–206.6] and 215.5% [175.1–262.1], respectively. HL demonstrated an increase in incident cases, no change in deaths, and a decrease in DALYs. For ASRs (Table 1, Figure 7D–F), MM experienced the largest relative percentage increases of 86.8% [61.5–112.0] in ASIR, 60.6% [42.3–78.6] in ASMR, and 56.5% [39.4–72.6] in ASDALYR. Leukemia demonstrated increases in ASIR and ASMR; NHL demonstrated increases in ASIR but decreases in ASDALYR; and HL demonstrated decreases in ASMR and ASDALYR. In terms of sex from 1990 to 2021, the male-to-female ratio for ASIR, ASMR, and ASDALYR increased for leukemia and HL but decreased for MM (Figure 7G–I). For NHL, ASIR and ASMR increased, while ASDALYR did not show any significant trends. Among all age groups in 2021 (Supplementary Figure S15A–C), the most incident cases occurred in the 65–69 group for leukemia, MM, and NHL compared to the 60–64 group for HL. The most deaths occurred in the 65–69 group for leukemia, MM, and HL compared to the 80–84 group for NHL. The most DALYs occurred in leukemia, MM, and NHL compared to the 55–59 group for HL.

From 1990 to 2021, incident cases increased for all HMs, while deaths and DALYs increased for AML, CLL, and other leukemia (Supplementary Table S1, Supplementary Figure S16A–C). The largest increases in incident cases, deaths, and DALYs occurred in AML, CLL, and other leukemia. While CLL demonstrated the largest relative percentage increases of around 200–500%, AML demonstrated the largest absolute increases in total cases. For ASIR, all HMs demonstrated relative percentage increases except for CML, with the largest increase in CLL of 152.8% [83.4–246.7] (Supplementary Table S1, Supplementary Figure S16D). For ASMR, only AML demonstrated a relative percentage change, increasing by 38.9% [18.1–62.0] (Supplementary Table S1, Supplementary Figure S16E). For ASDALYR, AML and CLL increased, while CML decreased (Supplementary Table S1, Supplementary Figure S16F). In terms of sex from 1990 to 2021, the male-to-female ratio for ASIR, ASMR, and ASDALYR increased for all leukemia subtypes (Supplementary Figure S16G–I). Among all age groups in 2021 (Supplementary Figure S15D–F), the most incident cases, deaths, and DALYs occurred in the 65–69 group for all leukemia subtypes, except ALL, with the most incident cases in the <5 group and the most DALYs in the 55–59 group.

## 4. Discussion

### 4.1. East Asia

While prior research studies have investigated the global burden of HMs and the burden of cancer in Asia [5,21,22], the findings presented in this report represent the first focused, systematic evaluation of the burden of HMs in East Asia from 1990 to 2021. East Asia accounts for a substantial proportion of the global burden of HMs due to the burden from Japan and China, with China having the highest global burden of HMs. Similar to previously reported global trends [5], all significant changes in the incident cases and deaths of leukemia, MM, NHL, and HL in East Asian countries from 1990 to 2021 were increases, with the most significant increases in MM and NHL. Total DALYs increased for both NHL and MM while having variable changes for leukemia and HL. The increase in East Asia leukemia incident cases and deaths was driven by significant increases in CLL incident cases and deaths.

From 1990 to 2021, ASIR in most HMs and countries increased while ASMR and ASDALYR in most decreased, which is consistent with previously reported global decreases in ASMR in HMs [5] and perhaps reflective of improved cancer health systems infrastructure leading to more diagnoses and modern therapeutics improving mortality and morbidity [23,24]. Medical advances, such as the widespread adoption of hematopoietic stem cell transplantation, targeted therapies, and immunotherapies, have substantially improved outcomes over the past three decades [25]. The notable exception in East Asia is the significant increase of MM ASIR, ASMR, and ASDALYR in Mongolia, Taiwan, and especially China, with increases between 200 and 300% across ASRs. From 1990 to 2021, ASIR in leukemia subtypes decreased in CML, increased in CLL, and had variable changes in the other subtypes. ASMR and ASDALYR decreased in most countries and in leukemia subtypes.

Aside from MM in North Korea and some outliers for leukemia subtypes, males consistently had higher ASIR, ASMR, and ASDALYR from 1990 to 2021. Previous epidemiological studies in HMs have shown higher ASIR in males compared to females, which has been attributed to various factors, including sex differences in hormones, genetics, environmental exposure, and lifestyle [5,26,27]. The effect of sex on mortality and morbidity needs to be further investigated. Since 1990, there have been dramatic decreases in deaths and DALYs in children, especially in leukemia. With these improvements in pediatric mortality and morbidity, combined with the aging population, the weight of HM burden has shifted even more towards older adults. Improvements in the pediatric population can largely be attributed to the dramatic decrease in childhood leukemia deaths and DALYs in China.

The large absolute increases over time in NHL and MM cases are likely attributed to a combination of population growth, the world’s aging population, better diagnostic capabilities, changing diagnostic criteria, and the rising global rates of obesity [28]. The aging population can only account for part of the increase in NHL and MM cases, as evidenced by the persistent increases over time after age-standardization. For MM, the updating of the MM diagnostic criteria with new myeloma-defining events, including laboratory and imaging findings beyond the traditional CRAB features indicating end-organ damage, may have led to increased diagnosis [29]. For lymphoma, the addition of immunophenotyping, cytogenetics, and molecular criteria may have also led to diagnosis of previously uncharacterized lymphoma subtypes [30]. Furthermore, obesity has been shown to be linked to the risk of developing MM and NHL and to worse outcomes [31,32,33].

However, great heterogeneity exists among East Asian countries, which have unique socio-political and cultural challenges. The following sections explore the country-specific context of our findings.

### 4.2. China

China has the largest global burden of HMs, reflecting its massive population. NHL and MM experienced large absolute increases across all measures. One of the most notable findings was the tripling of MM ASIR and the doubling of MM ASMR and ASDALYR in China, which were the largest changes noted in ASRs in all East Asian countries and HMs. Similar to what was previously discussed for East Asia as a whole, these increases are potentially driven by population growth, an aging population, better diagnostic capabilities, changing diagnostic criteria, and the rising rates of obesity. China has one of the fastest aging populations due to increases in life expectancy and declines in fertility rates [34]. China has also experienced significant increases in obesity prevalence and has a large population of overweight or obese individuals, more than that of India and the USA combined [28]. Worsening ASMR and ASDALYR suggest that despite the vast improvements in MM treatment due to autologous stem cell transplantation and the introduction of novel agents in the early 21st century, MM remains a significant challenge in China. China has also experienced a dramatic decrease in deaths and DALYs in pediatric leukemia, specifically ALL. These improvements are reflective of the dramatic advances in treatment of pediatric ALL over the last couple of decades [35]. However, despite these improvements, one in every five children with ALL globally is in China [36], highlighting the persistent burden of childhood leukemia.

China faces unique challenges in cancer control and healthcare access more broadly. While China implemented a national Basic Public Health Service (BPHS) program in 2009, which provided free basic health services for all residents, many inequities still exist that prevent patients from having access or being able to afford cancer treatment, including but not limited to urban–rural and resource-rich/resource-poor region disparities [37]. Chinese government initiatives and policies, including the establishment of a cancer registration framework across nearly all counties after becoming a collaborating center of the Global Initiative for Cancer Registry Development in 2017, Healthy China 2030, and the Healthy China Action Plan (2019–2030), are important in improving cancer care and reducing disparities in China [38,39].

### 4.3. Japan

Japan contributes considerably to the global burden of hematologic cancers, in part also due to its large population. Furthermore, absolute incidence, mortality, and morbidity associated with these cancers in Japan continue to rise, particularly for MM and NHL; age-standardized incidence also increased for NHL and HL. Absolute increases are likely driven by Japan’s aging population [40], underscoring the need to continue to invest in the development and maintenance of cancer systems in the country. Corollary to the aging population may be the lower proportion of younger patients with HMs who may benefit from transplantation; one study suggests that the demand in Japan for myeloablative transplantation will decrease from 2010 to 2040 by 67% [40].

Importantly, age-adjusted deaths and DALYs appear to be stable or decreasing, likely a testament to the strength of cancer care in Japan. As has been suggested for solid tumors, efforts are needed to invest in survivorship care for the growing number of cancer survivors [41]. Although financial toxicity remains a critical problem even in high-income settings like Japan [42], the Japanese healthcare system overall continues to be an exemplar of high-value care in the region [43]. Regional collaboration for cancer system strengthening may foster knowledge exchange specific to health services for patients with HMs.

### 4.4. North Korea

In general, information on the health status of the population, comprising approximately 26 million in North Korea, is largely unknown due to the paucity of data [44], unreliable sources [45], and limited access to the country itself [46]. The absence of significant changes in ASR for any HM may reflect the country’s limited capacity to diagnose these entities due to its hospitals’ lack of infrastructure and equipment [47,48]. Strikingly, new HM cases were diagnosed much earlier in North Korea compared to other East Asian countries, ranging from 5 to 20 years. This young age distribution is likely partially reflective of the shorter life expectancy of individuals in North Korea compared to other East Asian countries [49], with other country-specific mechanisms needing to be further investigated. However, it is important to note that significant changes have been made in the health sector since the start of the Kim Jong Un era [50], like the establishment of hospitals, medical research institutes, pharmaceutical factories, and research programs [50]. Hopefully, these reforms will improve the diagnostic and treatment landscape in the coming years.

### 4.5. Republic of Korea

As global population aging accelerates [51], Republic of Korea is among the fastest-aging countries worldwide [52]. The increasing proportion of elderly individuals in the population correlates with rising incident cases and an increase in DALYs for MM, as well as an increase in ASIR for NHL. However, the lack of a significant increase in MM incidence after age standardization suggests that additional factors are influencing these trends [53]. Advancements in medical technology have improved the diagnosis of HMs, contributing to earlier detection. Additionally, Republic of Korea’s national health insurance system, which covers 95% of cancer-related medical expenses, has significantly improved access to healthcare. Free national health checkups, provided to employees, the self-employed, and individuals receiving medical care, further promote early diagnosis by including tests such as complete blood cell counts [54,55,56]. A decline in the ASIR of leukemia has been observed in high-income Asian countries, possibly due to reductions in environmental risk factors and smoking, decreases in childhood leukemia, and increases in the intake of folate and vitamin supplementation during pregnancy [57,58]. Furthermore, increased availability of intensive treatment for elderly patients [59], combined with Republic of Korea’s expansion of reimbursed treatments for older leukemia patients [57], may explain the declining ASMR for leukemia except for AML which continues to have poor outcomes in older patients [60].

### 4.6. Mongolia

In light of an epidemiological transition from 1990 to 2019, the burden of disease in Mongolia, now home to 3.4 million people [61], shifted toward non-communicable diseases, including cancer (and, in particular, cancers of the liver, lung, esophagus, and stomach) [62]. Although less common than these solid tumors, HMs in Mongolia are on the rise as well, particularly MM, NHL, and HL. These findings may be due to improved diagnostic capabilities [63] and decreases in the competing risks of non-cancer causes of morbidity and mortality. Similar to North Korea, new cases of HMs were diagnosed at a significantly younger age compared to other East Asian countries due to the shorter life expectancy of Mongolian individuals [61].

Since the 1990s, Mongolia has experienced significant increases in life expectancy [61], likely due to the improved access and quality of healthcare, hygiene, and living conditions, as well as the implementation of various national policies and programs [62]. Notably, the health insurance law passed in 1993, which introduced social health insurance as part of a larger social security system, resulted in over 90% coverage by 2019 [62]. Recent efforts have sought to implement population-level screening for non-communicable diseases in the country [64]. For HMs specifically, recent advances are promising: the first autologous stem cell transplantation was performed in 2014, and the first allogenic stem cell transplantation was performed in 2020 [65]. However, the majority of cancer services (surgical and radiation services) are still centralized in the nation’s capital [64], underscoring the need for multidisciplinary approaches to tackling barriers in access to cancer care.

### 4.7. Taiwan

The absolute counts of incidence, deaths, and DALYs increased for leukemia, MM, and NHL in Taiwan. Similar to China and Mongolia, the ASRs for MM all increased over time. Paralleling the rising burden of HMs in Taiwan is also the improvement of therapeutic options, such as hematopoietic stem cell transplantation, largely due to the leadership and guidance of the Taiwan Society of Blood and Marrow Transplantation, formed in 1992 [66]. However, Taiwan faces similar challenges to other East Asian countries, including a significant increase in obesity prevalence over time [28] and a rapidly aging population [67], both of which are contributing to the rising HM burden in Taiwan. Overall, Taiwanese citizens have had great healthcare access, enjoying universal healthcare since 1995. The Taiwan National Healthcare Insurance program covers 99% of citizens and reimburses most Taiwan Food and Drug Administration-approved drugs [68]. However, low insurance premiums have led to financial challenges, resulting in cost-cutting measures such as long working hours and low salaries [69]. These challenges, combined with the increasingly aging population, are placing a significant burden on the Taiwanese healthcare system [69].

## 5. Limitations

Limitations of our study align with previously described deficiencies in prior GBD studies [18,19,20]. Data and estimations generated by the GBD study depend on the quality of the data. Specifically, in East Asia, North Korea does not have any cancer data registries, Republic of Korea has many years of incidence data but no mortality data, and Mongolia has only incidence data from 2003–2007 but no mortality data. Better reporting quality may result in overestimation, while underreporting may result in underestimation. GBD 2021 also did not account for the impact of the COVID-19 pandemic on cancer estimates. Estimates reported here should be interpreted with these limitations in mind.

## 6. Conclusions

Given the significant increasing burden of HMs in China and Japan, diagnosis and treatment of HMs in those countries should be a primary global health focus, especially NHL and MM. Significant variations between the other East Asian countries also warrant further country- and disease-specific investigations.

## Figures and Tables

**Table 1:** Hematological malignancy burden by country, Both sexes.

Location	Incidence				Deaths				DALYs			
Hematological Malignancy	Counts, 2021	Percent change, 1990–2021	ASR, 2021	Percent change, 1990–2021	Counts, 2021	Percent change, 1990–2021	ASR, 2021	Percent change, 1990–2021	Counts, 2021	Percent change, 1990–2021	ASR, 2021	Percent change, 1990–2021
**East Asia**												
Leukemia	125,491.3	40.8%	**··**	**··**	73,914.8	-4.0%	**··**	**··**	2,553,059.9	−40.8%	**··**	**··**
Multiple Myeloma	27,838.3	375.2%	**··**	**··**	20,539.4	330.7%	**··**	**··**	471,351.5	291.3%	**··**	**··**
Non-Hodgkin Lymphoma	155,393.9	246.4%	**··**	**··**	62,706.2	97.0%	**··**	**··**	1,660,918.3	39.4%	**··**	**··**
Hodgkin Lymphoma	5093.3	−2.2%	**··**	**··**	2797.9	−39.8%	**··**	**··**	83,423.8	−56.4%	**··**	**··**
**China**												
Leukemia	105,667.2 [75,275.7–132,236.9]	**38.7** **[7.6–82.9]**	7.2 [4.9–9.1]	0.9 [−23.3 to 35.1]	58,903.5 [43,626.0–74,038.9]	−12.6 [−29.1 to 14.6]	3.4 [2.5–4.3]	**−47.0** **[−56.7 to −31.4]**	2,205,220.6 [1,612,838.7–2,736,625.1]	**−43.8** **[−55.3 to −25.6]**	151.5 [108.7–185.1]	**−55.9** **[−65.3 to −41.6]**
Multiple Myeloma	17,249.5 [11,016.7–22,663.0]	**918.7** **[310.4–1657.5]**	0.8 [0.5–1.1]	**312.5** **[62.9–620.9]**	12,984.1 [8447.9–17,113.8]	**716.0** **[229.5–1314.1]**	0.6 [0.4–0.8]	**221.5** **[26.3–463.3]**	338,358.5 [213,668.7–447,634.9]	**622.1** **[192.7–1140.6]**	16.1 [10.1–21.3]	**221.1** **[28.5–459.2]**
Non-Hodgkin Lymphoma	110,923.5 [86,933.9–135,200.4]	**255.3** **[172.7–367.9]**	5.5 [4.4–6.7]	**66.7** **[28.7–117.0]**	42,856.9 [33,553.2–51,712.2]	**78.4** **[37.2–134.7]**	2.1 [1.7–2.6]	−20.1 [−38.0 to 3.8]	1,277,097.3 [997,263.0–1,551,799.7]	**33.1** **[1.1–74.5]**	66.9 [52.5–80.2]	**−27.6** **[−4.5 to −5.7]**
Hodgkin Lymphoma	4211.1 [2542.0–5541.5]	−11.3 [−39.7 to 57.3]	0.2 [0.1–0.3]	**−52.5** **[−67.8 to −13.6]**	2443.2 [1506.8–3231.6]	−44.4 [−62.6 to 0.4]	0.13 [0.08–0.17]	**−73.2** **[−81.8 to −50.3]**	74,190.6 [46,299.5–100,603.2]	**−59.2** **[−72.6 to −26.4]**	4.1 [2.6–5.6]	**−75.4** **[−83.5 to −54.6]**
**Japan**												
Leukemia	13,647.1 [12,049.6–14,607.2]	**46.7** **[33.6–56.8]**	5.7 [5.3–6.0]	**−16.9** **[−23.5 to −10.9]**	10,592.2 [9126.3–11,420.5]	**66.1** **[48.6–76.4]**	3.1 [2.8–3.2]	**−27.1** **[−31.1 to −24.5]**	206,496.8 [188,223.6–217,895.6]	**−8.1** **[−15.1 to −3.8]**	91.6 [87.1–94.7]	**−44.4** **[−46.3 to −42.8]**
Multiple Myeloma	7969.1 [6706.2–8881.7]	**118.1** **[92.5–141.8]**	2.0 [1.7–2.2]	−8.3 [−16.6 to 0.3]	5806.5 [4799.9–6373.2]	**111.2** **[87.1–125.7]**	1.3 [1.1–1.4]	**−20.0** **[−25.9. to −16.3]**	93,334.2 [80,942.2–101,130.6]	**51.0** **[37.0–59.5]**	25.7 [23.2–27.3]	**−28.4** **[−32.5 to −25.7]**
Non-Hodgkin Lymphoma	34,577.7 [29,092.6–39,491.1]	**210.5** **[165.7–261.9]**	10.4 [9.1–11.8]	**51.9** **[33.7–74.1]**	15,272.4 [12,775.5–16,644.2]	**157.9** **[127.1–174.4]**	3.6 [3.2–3.9]	0.07 [−7.3 to 4.3]	266,046.7 [234,467.9–287,122.6]	**66.0** **[50.3–75.4]**	84.7 [78.3–89.6]	**−16.5** **[−20.9 to −13.3]**
Hodgkin Lymphoma	507.7 [466.1–542.0]	**97.5** **[82.7–113.1]**	0.29 [0.27–0.32]	**69.4** **[55.3–84.4]**	236.0 [207.9–253.1]	**65.0** **[49.8–74.9]**	0.07 [0.07–0.08]	**−17.6** **[−21.8 to −14.0]**	5192.5 [4765.6–5515.2]	**13.9** **[6.3–19.8]**	2.4 [2.3–2.6]	**−20.0** **[−23.5 to −16.6]**
**North Korea**												
Leukemia	1414.1 [1019.5–1974.1]	40.1 [−0.6 to 95.3]	4.9 [3.5–6.8]	−4.8 [−33.6 to 33.8]	1201.2 [869.1–1689.8]	30.7 [−7.2 to 84.9]	4.1 [2.9–5.6]	−15.0 [−39.3 to 20.2]	50,535.6 [36,097.7–72,235.7]	1.0 [−29.2 to 45.0]	187.6 [134.5–265.5]	−21.5 [−45.4 to 11.8]
Multiple Myeloma	114.4 [64.9–191.9]	**124.5** **[42.2–239.1]**	0.3 [0.2–0.6]	11.1 [−29.0 to 66.0]	97.0 [54.2–163.6]	**112.4** **[33.3–218.7]**	0.3 [0.2–0.5]	2.7 [−34.3 to 53.5]	2705.5 [1534.7–4603.2]	**96.8** **[24.7–196.0]**	8.0 [4.6–13.7]	4.2 [−33.9 to 56.5]
Non-Hodgkin Lymphoma	1011.9 [732.3–1476.9]	**119.6** **[55.0–210.8]**	3.1 [2.3–4.5]	21.6 [−13.1 to 71.5]	789.8 [578.8–1145.4]	**93.5** **[34.8–171.9]**	2.4 [1.8–3.5]	3.1 [−27.3 to 44.5]	26,082.4 [18,935.8–37,918.2]	**65.6** **[14.5–140.0]**	81.6 [59.2–118.0]	0.7 [−29.3 to 45.4]
Hodgkin Lymphoma	106.3 [72.2–184.8]	62.8 [−3.0 to 164.5]	0.3 [0.2–0.6]	0.4 [−38.3 to 64.2]	49.5 [34.2–83.5]	21.5 [−24.8 to 92.6]	0.2 [0.1–0.3]	−32.1 [−57.2 to 6.5]	1762.7 [1183.3–3066.8]	2.1 [−40.7 to 66.8]	5.7 [3.9–9.9]	−34.3 [−61.2 to 6.8]
**Republic of Korea**												
Leukemia	3028.2 [1947.8–3706.7]	**52.4** **[2.3–99.9]**	4.8 [2.9–6.2]	−7.4 [−43.3 to 23.5]	1949.5 [1211.0–2389.6]	11.2 [−28.9 to 40.2]	2.4 [1.5–2.9]	**−48.5** **[−68.0 to −36.0]**	53,503.9 [34,349.8–64,911.7]	**−40.0** **[−57.6 to −19.7]**	82.0 [53.2–99.6]	**−60.5** **[−72.6 to −47.5]**
Multiple Myeloma	1672.6 [957.4–2207.4]	**487.7** **[187.5–758.6]**	1.8 [1.0–2.3]	73.8 [−18.3 to 159.4]	1100.9 [624.8–1455.9]	**334.2** **[111.9–539.5]**	1.2 [0.7–1.5]	22.7 [−41.5 to 85.1]	23,445.5 [13,793.7–30,965.4]	**247.4** **[82.6–413.5]**	24.7 [14.6–32.6]	13.6 [−41.2 to 64.2]
Non-Hodgkin Lymphoma	6167.4 [4153.5–7432.6]	**426.0** **[173.2–583.9]**	7.1 [4.9–8.5]	**111.7** **[2.6–173.9]**	2279.8 [1493.8–2707.5]	**163.8** **[27.1–233.0]**	2.5 [1.7–3.0]	−4.7 [−57.1 to 211.6]	53,856.1 [37,017.1–62,843.0]	59.6 [−8.6 to 96.9]	64.2 [45.2–74.4]	**−25.7** **[−58.9 to −8.8]**
Hodgkin Lymphoma	132.5 [77.3–185.9]	**125.2** **[30.0–242.5]**	0.2 [0.1–0.3]	40.1 [−16.0 to 112.9]	42.4 [23.8–58.9]	−4.5 [−44.2 to 43.6]	0.05 [0.03–0.07]	**−59.3** **[−75.1 to −38.8]**	1247.8 [717.0–1745.4]	**−34.2** **[–62.1 to –1.3]**	1.8 [1.0–2.5]	**−60.6** **[−76.8 to −41.0]**
**Mongolia**												
Leukemia	79.5 [59.1–100.4]	29.7 [−4.4 to 79.0]	2.7 [2.0–3.3]	−19.9 [−39.2 to 7.2]	71.0 [52.8–90.0]	19.8 [−10.5 to 63.5]	2.4 [1.8–3.1]	−24.8 [−43.1 to 0.5]	3512.4 [2590.3–4496.3]	−4.4 [–31.4 to 31.2]	108.1 [80.4–137.4]	**−32.6** **[−50.2 to −7.5]**
Multiple Myeloma	8.0 [5.6–10.8]	**312.9** **[182.7–547.0]**	0.3 [0.2–0.4]	**77.1** **[23.6–173.9]**	7.1 [5.0–9.7]	**279.0** **[165.2–490.4]**	0.3 [0.2–0.4]	**66.5** **[18.0–157.6]**	238.9 [168.1–328.8]	**295.7** **[172.3–523.7]**	8.4 [5.9–11.5]	**68.9** **[17.6–164.4]**
Non-Hodgkin Lymphoma	61.9 [46.9–81.6]	**56.7** **[3.2–142.9]**	2.1 [1.6–2.8]	−14.9 [−43.1 to 31.8]	36.4 [27.7–48.3]	16.8 [−21.9 to 77.7}	1.3 [1.0–1.8]	**−35.5** **[−56.8 to −1.9]**	1526.1 [1169.5–2036.5]	−6.5 [–38.0 to 46.0]	49.1 [37.6–65.4]	**−41.6** **[−60.6 to −10.2]**
Hodgkin Lymphoma	18.7 [11.2–27.3]	**137.9** **[34.8–379.4]**	0.6 [0.4–0.9]	25.1 [−30.0 to 150.9]	10.3 [6.2–14.9]	63.6 [−9.5 to 229.6]	0.3 [0.2–0.5]	−13.4 [−52.6 to 75.6]	478.1 [291.3–696.8]	45.5 [–17.4 to 193.5]	14.8 [9.0–21.7]	−17.3 [−53.1 to 63.7]
**Taiwan**												
Leukemia	1655.2 [1496.0–1798.8]	**178.3** **[148.6–206.6]**	5.1 [4.7–5.6]	**55.7** **[38.9–73.8]**	1197.2 [1090.9–1290.6]	**144.1** **[120.7–165.8]**	3.3 [3.0–3.5]	**16.2** **[5.5–26.2]**	33,790.7 [30,979.9–36,420.1]	**51.5** **[37.5–65.3]**	114.2 [105.1–123.1]	0.3 [−8.4 to 8.9]
Multiple Myeloma	824.7 [724.5–935.7]	**374.5** **[304.7–439.6]**	2.0 [1.8–2.2]	**86.8** **[61.5–112.0]**	543.9 [490.1–598.1]	**329.1** **[278.7–380.1]**	1.3 [1.2–1.4]	**60.6** **[42.3–78.6]**	13,268.9 [12,022.6–14,507.0]	**266.9** **[225.9–307.2]**	32.8 [29.8–35.7]	**56.5** **[39.4–72.6]**
Non-Hodgkin Lymphoma	2651.4 [2330.0–3005.4]	**215.5** **[175.1–262.1]**	7.0 [6.2–7.9]	**47.1** **[29.1–67.3]**	1470.8 [1312.5–1629.8]	**154.3** **[124.9–183.0]**	3.6 [3.3–4.0]	3.8 [−7.6 to 15.4]	36,309.7 [32,556.8–40,098.3]	**75.7** **[56.6–94.2]**	99.4 [89.8–109.5]	**−10.2** **[−19.7 to −0.8]**
Hodgkin Lymphoma	117.0 [98.6–137.9]	**56.3** **[25.0–91.7]**	0.4 [0.3–0.5]	2.5 [−18.0 to 28.1]	16.4 [14.1–19.0]	−14.7 [−27.7 to 0.7]	0.04 [0.04–0.05]	**−59.3** **[−65.6 to −51.9]**	552.1 [473.9–644.5]	**−33.3** **[−44.1 to −20.8]**	1.7 [1.4–2.0]	**−58.6** **[−65.7 to −50.5]**

The figures inside the square brackets represent the 95% uncertainty interval. Bolded numbers represent statistically significant percent changes. Data Source: Global Burden of Disease, Injuries and Risk Factors 2021 Study.

## Data Availability

Data are publicly available from the Global Burden of Disease 2021 Study.

## Supplementary Materials

The following supporting information can be downloaded at: https://www.mdpi.com/article/10.3390/jcm14238381/s1, Supplementary Table S1: Leukemia burden by location, Both sexes; Supplementary Figure S1: Maps of age-standardized rates of incidence, deaths, and DALYs per 100,000 in East Asia in 2021, both sexes; Supplementary Figure S2: Maps of age-standardized rates of incidence, deaths, and DALYs per 100,000 in East Asia in 2021, both sexes; Supplementary Figure S3: East Asia—Trends in Hematological Malignancy Burden from 1990 to 2021 by Sex; Supplementary Figure S4: East Asia—Trends in Leukemia Burden from 1990 to 2021 by Sex; Supplementary Figure S5: China—Hematological Malignancy Burden by Age, 1990 and 2021; Supplementary Figure S6: China—Trends in Leukemia Burden from 1990 to 2021; Supplementary Figure S7: Japan—Hematological Malignancy Burden by Age, 1990 and 2021; Supplementary Figure S8: Japan—Trends in Leukemia Burden from 1990 to 2021; Supplementary Figure S9: North Korea—Hematological Malignancy Burden by Age, 1990 and 2021; Supplementary Figure S10: North Korea—Trends in Leukemia Burden from 1990 to 2021; Supplementary Figure S11: Republic of Korea—Hematological Malignancy Burden by Age, 1990 and 2021; Supplementary Figure S12: Republic of Korea—Trends in Leukemia Burden from 1990 to 2021; Supplementary Figure S13: Mongolia—Hematological Malignancy Burden by Age, 1990 and 2021; Supplementary Figure S14: Mongolia—Trends in Leukemia Burden from 1990 to 2021; Supplementary Figure S15: Taiwan—Hematological Malignancy Burden by Age, 1990 and 2021; Supplementary Figure S16: Taiwan—Trends in Leukemia Burden from 1990 to 2021.
