# Supplementary material for: Burden of Hematological Malignancies in East Asia from 1990 to 2021
Source: J Clin Med. 2025 Nov 26;14(23):8381. doi: 10.3390/jcm14238381 (PMC12693399; doi:10.3390/jcm14238381)

List of Supplementary Tables and Figures

Supplementary Table S1: Leukemia burden by country, Both sexes.

Supplementary Figure S1: Maps of age-standardized rates of incidence, deaths, and DALYs per 100,000 in East Asia in 2021, both sexes.

Supplementary Figure S2: Maps of age-standardized rates of incidence, deaths, and DALYs per 100,000 in East Asia in 2021, both sexes.

Supplementary Figure S3: East Asia – Trends in Hematological Malignancy Burden from 1990 to 2021 by Sex.

Supplementary Figure S4: East Asia – Trends in Leukemia Burden from 1990 to 2021 by Sex.

Supplementary Figure S5: China – Hematological Malignancy Burden by Age, 1990 and 2021.

Supplementary Figure S6: China – Trends in Leukemia Burden from 1990 to 2021.

Supplementary Figure S7: Japan – Hematological Malignancy Burden by Age, 1990 and 2021.

Supplementary Figure S8: Japan – Trends in Leukemia Burden from 1990 to 2021.

Supplementary Figure S9: North Korea – Hematological Malignancy Burden by Age, 1990 and 2021.

Supplementary Figure S10: North Korea – Trends in Leukemia Burden from 1990 to 2021.

Supplementary Figure S11: South Korea – Hematological Malignancy Burden by Age, 1990 and 2021.

Supplementary Figure S12: South Korea – Trends in Leukemia Burden from 1990 to 2021.

Supplementary Figure S13: Mongolia – Hematological Malignancy Burden by Age, 1990 and 2021.

Supplementary Figure S14: Mongolia – Trends in Leukemia Burden from 1990 to 2021.

Supplementary Figure S15: Taiwan – Hematological Malignancy Burden by Age, 1990 and 2021.

Supplementary Figure S16: Taiwan – Trends in Leukemia Burden from 1990 to 2021.

Supplementary Table S1: Leukemia burden by country, Both sexes.

**Location**	**Incidence**				**Deaths**				**DALYs**			
Leukemia	Counts, 2021	Percent change, 1990−2021	ASR, 2021	Percent change, 1990−2021	Counts, 2021	Percent change, 1990−2021	ASR, 2021	Percent change, 1990−2021	Counts, 2021	Percent change, 1990−2021	ASR, 2021	Percent change, 1990−2021
**East Asia**												
Acute Myeloid Leukemia	27,390.0	35.1%	··	··	23,478.3	22.0%	··	··	730,315.7	−28.4%	··	··
Acute Lymphoid Leukemia	41,581.6	0.1%	··	··	22,360.3	−38%	··	··	988,901.7	−58.8%	··	··
Chronic Myeloid Leukemia	5570.2	−13.8%	··	··	2780.5	−46.5%	··	··	80,130.3	−65.9%	··	··
Chronic Lymphoid Leukemia	30,694.0	314.5%	··	··	9259.2	80.3%	··	··	281,388.9	52.8%	··	··
Other Leukemia	20,255.4	50.4%	··	··	16,036.4	41.3%	··	··	472,323.4	−0.1%	··	··
**China**												
Acute Myeloid Leukemia	17,835.2 [11876.2−24800.4]	16.5 [−31.1 to 100.9]	1.0 [0.7−1.4]	−29.6 [−57.2 to 15.1]	15,311.1 [10,365.3−21,401.3]	3.1 [−38.9 to 79.2]	0.9 [0.6−1.2]	−39.5 [−63.0 to 0.7]	548,555.4 [373,859.1−778,262.0]	−35.6 [−64.8 to 18.1]	37.0 [25.3−52.5]	**−50.8 [−73.4 to −11.1]**
Acute Lymphoid Leukemia	38,570.9 [21,149.3−50,762.3]	1.4 [−41.4 to 54.5]	3.6 [2.0−5.1]	7.5 [−40.2 to 66.8]	20,612.9 [11,781.4−27,301.8]	**−39.2 [−62.2 to −7.1]**	1.4 [0.8−1.7]	**−55.5 [−72.1 to −33.6]**	924,422.2 [525,901.7−1,182,319.5]	**−59.4 [−74.9 to −37.5]**	74.1 [42.9−95.0]	**−62.2 [−76.9 to −43.0]**
Chronic Myeloid Leukemia	3849.9 [2086.9−6105.4]	−1.7 [−39.3 to 77.0]	0.2 [0.1−0.3]	**−43.9 [−65.1 to −2.7]**	1840.7 [1080.2−3094.1]	**−48.7 [−69.0 to −4.4]**	0.10 [0.06−0.16]	**−72.0 [−82.9 to −49.8]**	60,928.7 [35,084.8−101,126.1]	**−65.7 [−79.4 to −35.4]**	3.6 [2.1−6.0]	**−76.6 [−85.8 to −56.5]**
Chronic Lymphoid Leukemia	28,926.7 [17,957.7−40,579.4]	**326.6 [215.2−503.5]**	1.4 [0.9−2.0]	**97.3 [48.4−176.1]**	8636.1 [5526.7−12,383.9]	**77.9 [32.6−149.1]**	0.4 [0.3−0.6]	−20.1 [−40.1 to 11.1]	268,250.5 [165,772.3−380,358.2]	**51.6 [11.3−117.0]**	14.1 [8.7−19.9]	−17.3 [−39.0 to 16.5]
Other Leukemia	16,484.5 [8183.5−23,107.5]	35.4 [−5.2 to 93.2]	0.9 [0.4−1.3]	−23.9 [−46.1 to 7.6]	12,502.7 [6546.7−17,075.4]	22.5 [−13.1 to 73.6]	0.6 [0.3−0.9]	**−38.3 [−54.9 to −14.5]**	403,063.8 [200,881.9−557,662.7]	−8.9 [−37.0 to 32.0]	22.8 [11.2−31.1]	**−43.1 [−60.0 to −20.3]**
**Japan**												
Acute Myeloid Leukemia	6506.4 [5635.0−7006.6]	**85.7 [66.4−99.1]**	2.0 [1.8−2.1]	**−10.8 [−16.2 to −6.1]**	5578.7 [4835.2−6001.0]	**84.7 [65.1−96.3]**	1.6 [1.5−1.7]	**−17.4 [−22.3 to −14.2]**	106,701.5 [96,598.3−112,648.2]	5.1 [−2.9 to 10.0]	45.7 [43.4−47.2]	**−36.2 [−38.4 to −34.4]**
Acute Lymphoid Leukemia	1694.9 [1573.2−1830.7]	**−25.4 [−36.0 to −14.7]**	2.0 [1.8−2.2]	−16.7 [−31.9 to 0.4]	890.6 [802.7−946.6]	**−18.4 [−25.2 to −13.9]**	0.43 [0.41−0.45]	**−49.3 [−51.0 to −47.7]**	26,439.8 [25,078.7−27,389.8]	**−52.1 [−54.4 to −50.3]**	21.8 [21.0−22.7]	**−55.6 [−57.3 to −53.9]**
Chronic Myeloid Leukemia	1145.3 [979.0−1272.8]	**−44.2 [−50.9 to −37.9]**	0.43 [0.38−0.48]	**−67.7 [−71.3 to −64.0]**	656.3 [536.0−737.5]	**−45.1 [−53.5 to −40.0]**	0.16 [0.14−0.18]	**−79.3 [−81.0 to −77.9]**	10,864.1 [9464.7−11,908.5]	**−72.3 [−75.3 to −70.1]**	4.4 [4.1−4.7]	**−83.7 [−84.5 to −82.8]**

Chronic Lymphoid Leukemia	1056.5 [917.9−1150.9]	**134.4 [111.8−154.2]**	0.28 [0.25−0.31]	**8.0 [0.6−15.9]**	368.7 [304.4−406.7]	**117.1 [91.2−134.1]**	0.07 [0.06−0.08]	**−27.6 [−33.6 to −23.3]**	6043.2 [5141.0−6657.1]	**62.5 [46.1−74.1]**	1.6 [1.4−1.7]	**−29.3 [−34.1 to −25.2]**
Other Leukemia	3244.0 [2791.3−3531.7]	**217.5 [188.8−243.8]**	0.97 [0.86−1.06]	**58.6 [45.9−70.9]**	3097.9 [2673.8−3383.3]	**244.3 [211.4−267.1]**	0.79 [0.70−0.85]	**48.6 [39.5−55.9]**	56,448.1 [50,523.8−60,759.8]	**125.2 [107.1−137.6]**	18.1 [16.7−19.2]	**21.4 [15.7−26.2]**
**North Korea**												
Acute Myeloid Leukemia	399.5 [250.4−719.8]	28.7 [−15.6 to 96.6]	1.3 [0.9−2.4]	−15.8 [−44.5 to 26.3]	382.2 [241.2−682.8]	26.9 [−17.6 to 92.3]	1.3 [0.8−2.3]	−18.3 [−45.6 to 21.9]	15,513.5 [9730.6−27,498.6]	−6.2 [−41.2 to 49.0]	57.0 [36.5−97.8]	−27.5 [−53.9 to 13.0]
Acute Lymphoid Leukemia	518.0 [262.4−760.0]	20.3 [−19.2 to 77.0]	2.0 [1.0−2.9]	−4.7 [−37.7 to 45.0]	465.4 [238.0−688.4]	18.1 [−20.4 to 74.0]	1.7 [0.9−2.4]	−14.6 [−42.8 to 27.3]	22,082.9 [11,578.5−32,166.0]	−8.1 [−39.2 to 38.2]	89.4 [45.6−134.0]	−21.0 [−49.2 to 20.2]
Chronic Myeloid Leukemia	90.6 [44.4−179.2]	22.5 [−39.6 to 98.0]	0.3 [0.1−0.6]	−25.7 [−63.8 to 20.9]	72.1 [36.2−141.5]	5.7 [−46.8 to 70.9]	0.2 [0.1−0.4]	−37.3 [−68.7 to 0.4]	2863.8 [1411.6−5637.7]	−10.5 [−55.6 to 46.2]	9.6 [4.8−18.7]	−38.9 [−69.5 to 1.3]
Chronic Lymphoid Leukemia	199.7 [107.4−326.2]	**165.3 [55.6−314.8]**	0.6 [0.3−1.0]	43.7 [−12.6 to 124.5]	108.8 [58.3−170.8]	**102.6 [23.4−212.4]**	0.3 [0.2−0.5]	5.5 [−32.3 to 64.6]	3700.5 [1905.5−5934.6]	**88.3 [5.7−207.3]**	11.4 [5.8−18.5]	10.8 [−37.8 to 81.0]
Other Leukemia	206.4 [118.3−354.0]	**72.6 [12.8−162.8]**	0.6 [0.4−1.1]	3.8 [−31.1 to 57.6]	172.7 [99.3−282.3]	**69.4 [13.6−160.1]**	0.5 [0.3−0.9]	−4.4 [−35.2 to 46.1]	6374.9 [3674.3−10,636.2]	48.3 [−3.1 to 132.2]	20.2 [11.7−33.4]	−4.9 [−37.9 to 48.1]
**South Korea**												
Acute Myeloid Leukemia	1688.6 [819.8−2146.2]	103.1 [−9.8 to 168.4]	2.1 [1.0−2.6]	−6.3 [−59.2 to 26.1]	1357.3 [656.2−1722.0]	74.0 [−24.6 to 131.9]	1.6 [0.8−2.0]	−24.2 [−67.8 to 2.2]	35,415.2 [18,178.5−43,618.0]	−4.4 [−52.7 to 25.1]	49.9 [26.3−61.2]	**−42.9 [−72.0 to −26.0]**
Acute Lymphoid Leukemia	562.8 [281.2−790.6]	−13.7 [−56.0 to 44.1]	1.8 [0.8−2.7]	17.3 [−43.3 to 96.3]	240.5 [123.7−364.2]	**−57.0 [−76.0 to −26.7]**	0.4 [0.2−0.6]	**−69.2 [−83.0 to −51.1]**	9786.6 [5017.0−13,691.4	**−72.4 [−84.8 to −53.3]**	21.8 [11.6−29.0]	**−72.2 [−85.0 to −53.8]**
Chronic Myeloid Leukemia	320.7 [166.2−568.5]	−4.1 [−40.7 to 83.6]	0.4 [0.2−0.7]	**−51.4 [−68.5 to −12.1]**	124.6 [66.6−218.9]	**−54.7 [−71.7 to −14.4]**	0.2 [0.1−0.3]	**−80.0 [−87.0 to −65.7]**	3184.3 [1671.2−5600.1]	**−74.5 [−84.2 to −48.2]**	4.5 [2.4−8.0]	**−84.4 [−90.0 to −69.8]**
Chronic Lymphoid Leukemia	264.0 [160.2−466.6]	**354.5 [161.4−670.9]**	0.3 [0.2−0.5]	33.9 [−20.3 to 114.9]	72.1 [43.9−119.9]	**95.4 [16.4−211.8]**	0.08 [0.05−0.13]	**−48.3 [−68.4 to −20.9]**	1484.9 [905.7−2733.7]	56.0 [−11.6 to 171.1]	1.6 [1.0−2.9]	**−50.4 [−71.4 to −17.2]**
Other Leukemia	192.1 [100.6−334.4]	72.6 [−3.2 to 173.9]	0.2 [0.1−0.4]	**−37.7 [−64.6 to −0.9]**	155.1 [81.6−272.4]	51.7 [−16.5 to 141.1]	0.2 [0.1−0.3]	**−50.2 [−72.5 to −20.5]**	3632.9 [1959.4−6228.2]	11.1 [−37.2 to 73.6]	4.1 [2.2−7.1]	**−56.1 [−75.0 to −31.0]**
**Mongolia**												
Acute Myeloid Leukemia	38.5 [26.4−51.3]	**73.0 [1.9−195.3]**	1.3 [0.9−1.7]	−1.0 [−36.9 to 66.3]	36.3 [25.0−48.5]	66.3 [−1.4 to 181.4]	1.2 [0.9−1.6]	−3.6 [−38.4 to 61.9]	1731.1 [1159.9−2306.9]	36.4 [−25.1 to 142.0]	53.4 [36.2−71.5]	−10.4 [−46.7 to 54.2]
Acute Lymphoid Leukemia	24.1 [15.2−33.2]	−16.6 [−50.0 to 26.5]	0.7 [0.5−1.0]	**−40.4 [−63.0 to −11.7]**	21.5 [13.5−29.1]	−23.3 [−52.4 to 14.2]	0.7 [0.4−0.9]	**−44.2 [−64.9 to −18.3]**	1296.6 [812.4−1757.7]	**−35.7 [−61.2 to −1.8]**	38.2 [23.8−51.7]	**−49.5 [−68.7 to −24.7]**
Chronic Myeloid Leukemia	5.4 [2.9−8.5]	45.4 [−28.6 to 210.6]	0.2 [0.1−0.3]	−24.7 [−63.6 to 65.5]	4.5 [2.4−7.2]	25.4 [−37.6 to 173.6]	0.2 [0.1−0.3]	−32.8 [−66.5 to 46.0]	177.8 [95.0−284.0]	0.6 [−51.7 to 110.8]	5.8 [3.1−9.2]	−40.8 [−70.8 to 27.5]
Chronic Lymphoid Leukemia	5.2 [2.8−8.7]	**140.8 [32.2−345.9]**	0.2 [0.1−0.3]	3.6 [−41.8 to 88.6]	3.2 [1.7−5.3]	70.7 [−4.3 to 214.8]	0.14 [0.07−0.22]	−22.2 [−56.0 to 43.1]	105.6 [56.9−171.6]	80.4 [−1.9 to 251.9]	3.8 [2.0−6.2]	−22.2 [−56.7 to 45.1]

Other Leukemia	6.4 [3.3−10.0]	48.1 [−13.4 to 130.3]	0.2 [0.1−0.4]	−26.7 [−55.6 to 12.2]	5.5 [2.9−8.5]	40.2 [−15.0 to 117.0]	0.2 [0.1−0.3]	−30.9 [−58.3 to 6.5]	201.3 [105.3−319.1]	29.8 [−24.4 to 110.2]	6.9 [3.7−10.9]	−34.6 [−61.5 to 4.3]
**Taiwan**												
Acute Myeloid Leukemia	921.9 [835.5−1004.0]	**207.8 [160.8−266.3]**	2.6 [2.3−2.8]	**53.7 [29.7−81.7]**	812.7 [732.7−879.7]	**194.8 [151.8−247.9]**	2.2 [2.0−2.4]	**38.9 [18.1−62.0]**	22,398.9 [20,670.8−24,153.1]	**83.0 [54.7−120.1]**	74.2 [68.5−79.8]	**18.3 [0.4−40.4]**
Acute Lymphoid Leukemia	210.9 [182.3−241.3]	**53.7 [12.0−125.1]**	1.2 [1.0−1.5]	**71.3 [17.1−168.1]**	129.5 [116.3−142.5]	27.4 [−3.0 to 80.2]	0.44 [0.40−0.48]	−16.2 [−35.2 to 18.3]	4873.6 [4407.8−5402.8]	−17.2 [−36.8 to 16.7]	22.0 [19.9−24.8]	−23.9 [−41.8 to 8.2]
Chronic Myeloid Leukemia	158.3 [128.8−194.9]	**97.0 [25.4−285.7]**	0.43 [0.36−0.53]	−4.4 [−39.4 to 83.4]	82.3 [67.7−100.3]	34.6 [−11.3 to 157.9]	0.21 [0.18−0.25]	−43.2 [−62.6 to 7.0]	2111.8 [1764.5−2555.5]	−13.0 [−45.3 to 70.9]	6.2 [5.3−7.5]	**−50.2 [−68.6 to −4.7]**
Chronic Lymphoid Leukemia	242.0 [205.3−288.2]	**531.9 [362.9−768.1]**	0.6 [0.5−0.7]	**152.8 [83.4−246.7]**	70.2 [58.4−84.0]	**287.4 [183.3−430.3]**	0.17 [0.14−0.20]	33.2 [−2.6 to 82.8]	1804.2 [1508.7−2143.3]	**229.8 [142.9−343.6]**	4.6 [3.9−5.4]	**42.9 [4.9−92.9]**
Other Leukemia	122.1 [95.0−149.1]	**210.3 [128.1−318.1]**	0.33 [0.25−0.40]	**42.3 [4.6−90.5]**	102.6 [79.0−122.8]	**202.4 [125.4−309.1]**	0.25 [0.20−0.30]	21.1 [−9.3 to 62.3]	2602.3 [2056.8−3103.1]	**115.1 [59.8−188.3]**	7.1 [5.7−8.5]	9.4 [−17.9 to 46.5]

The figures inside the square brackets represent the 95% uncertainty interval. Bolded numbers represent statistically significant percent changes. Data Source: Global Burden of Disease, Injuries and Risk Factors 2021 Study.

Supplementary Figure S1: Maps of age-standardized rates of incidence, deaths, and DALYs per 100,000 in East Asia in 2021, both sexes. (A) Leukemia ASIR. (B) Leukemia ASMR. (C) Leukemia ASDALYR. (D) MM ASIR. (E) MM ASMR. (F) MM ASDALYR. (G) NHL ASIR. (H) NHL ASMR. (I) NHL ASDALYR. (J) HL ASIR. (K) HL ASMR. (L) HL ASDALYR.

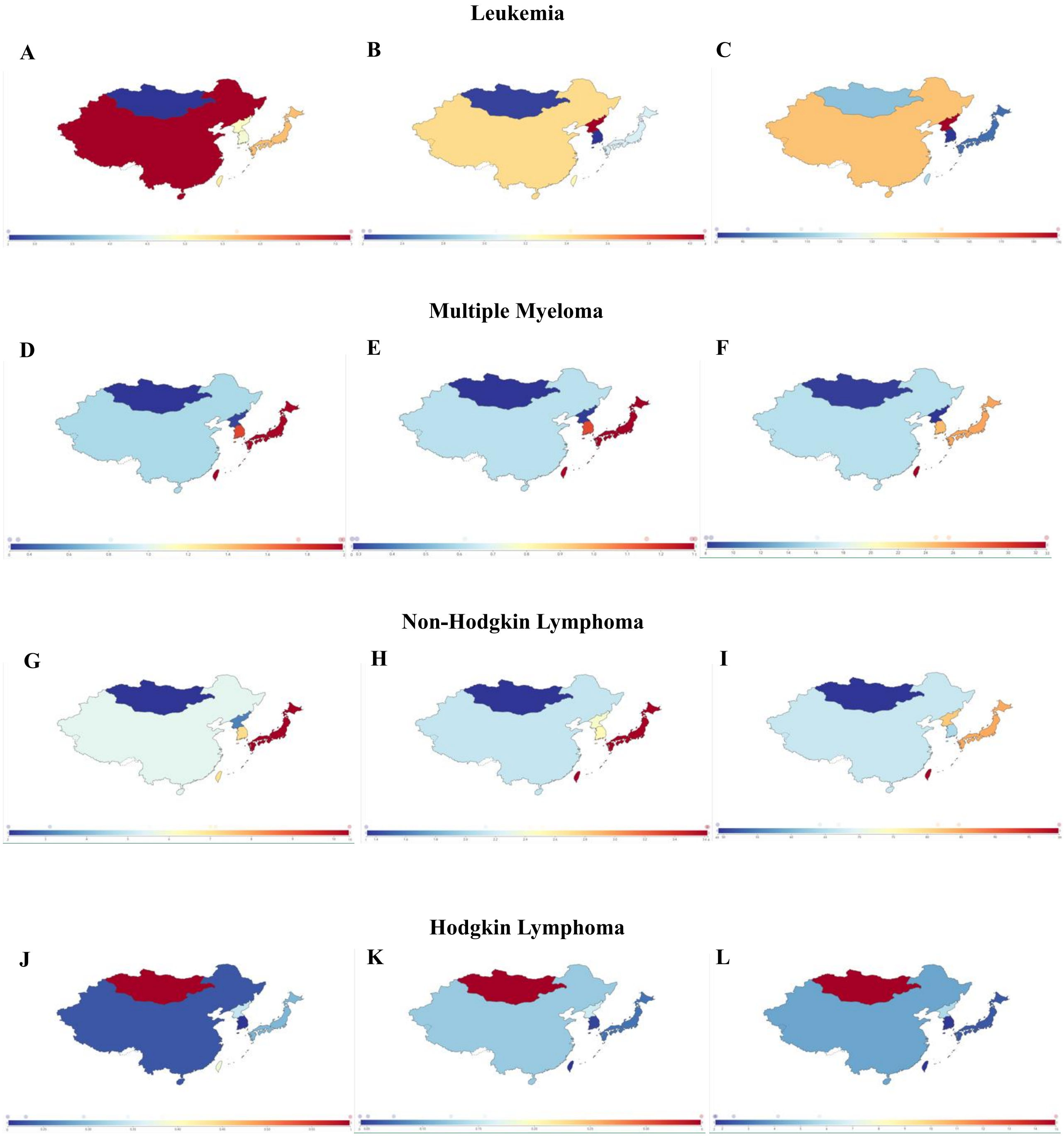

Supplementary Figure S2: Maps of age-standardized rates of incidence, deaths, and DALYs per 100,000 in East Asia in 2021, both sexes. (A) AML ASIR. (B) AML ASMR. (C) AML ASDALYR. (D) ALL ASIR. (E) ALL ASMR. (F) ALL ASDALYR. (G) CML ASIR. (H) CML ASMR. (I) CML ASDALYR. (J) CLL ASIR. (K) CLL ASMR. (L) CLL ASDALYR. (M) Other leukemia ASIR. (N) Other leukemia ASDR. (O) Other leukemia ASDALYR.

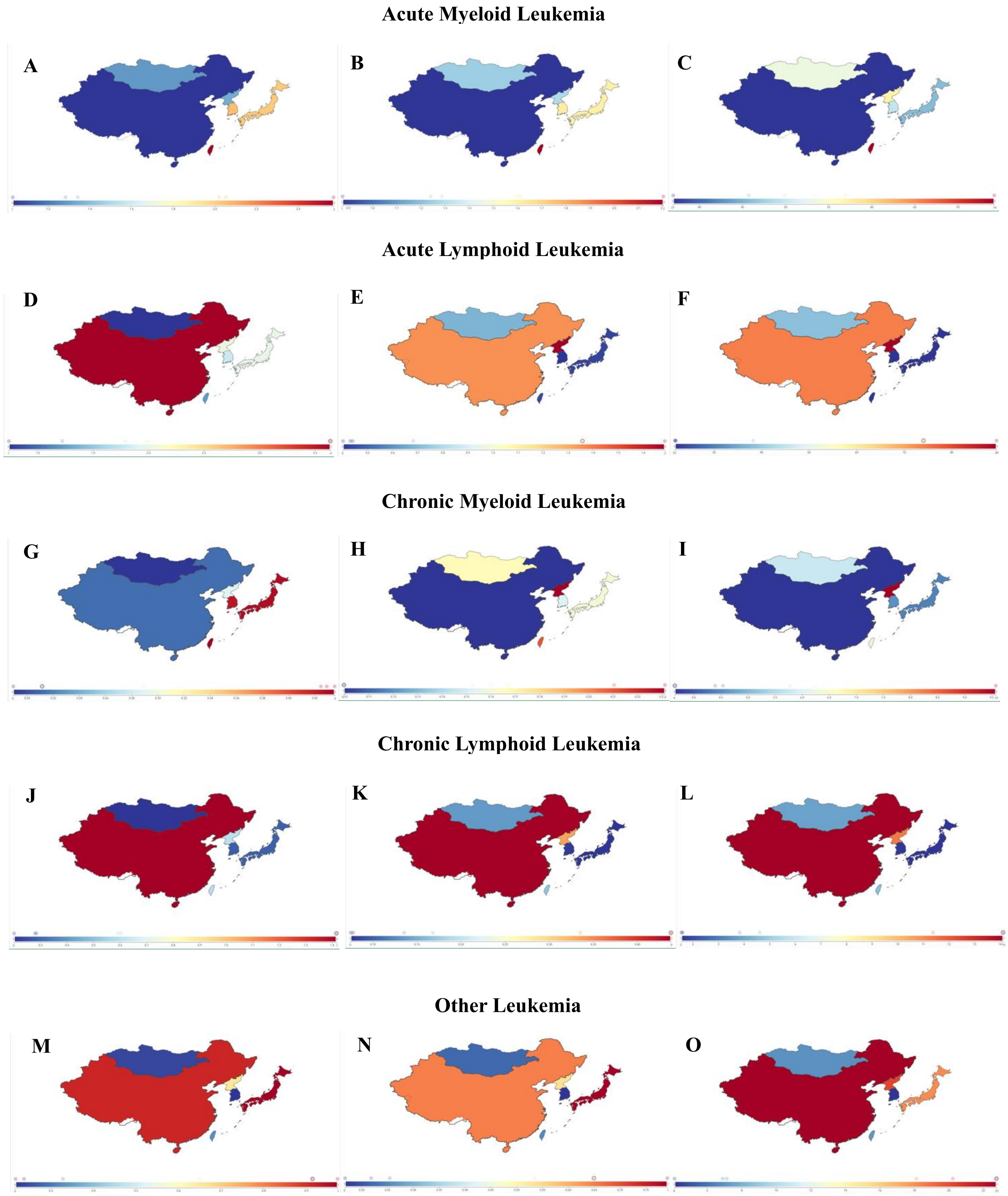

Supplementary Figure S3: East Asia – Trends in Hematological Malignancy Burden from 1990 to 2021 by Sex.

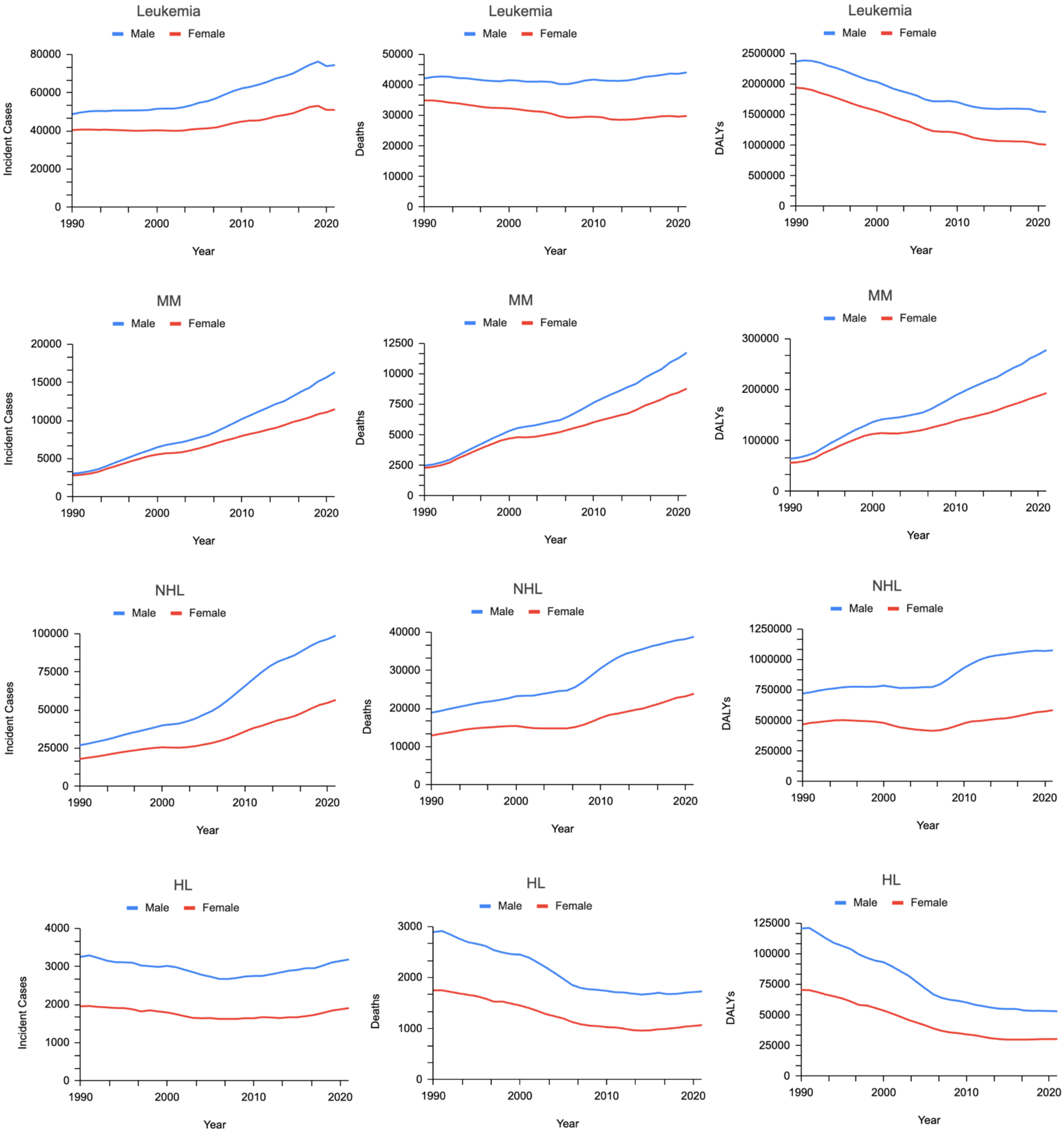

Supplementary Figure S4: East Asia – Trends in Leukemia Burden from 1990 to 2021 by Sex.

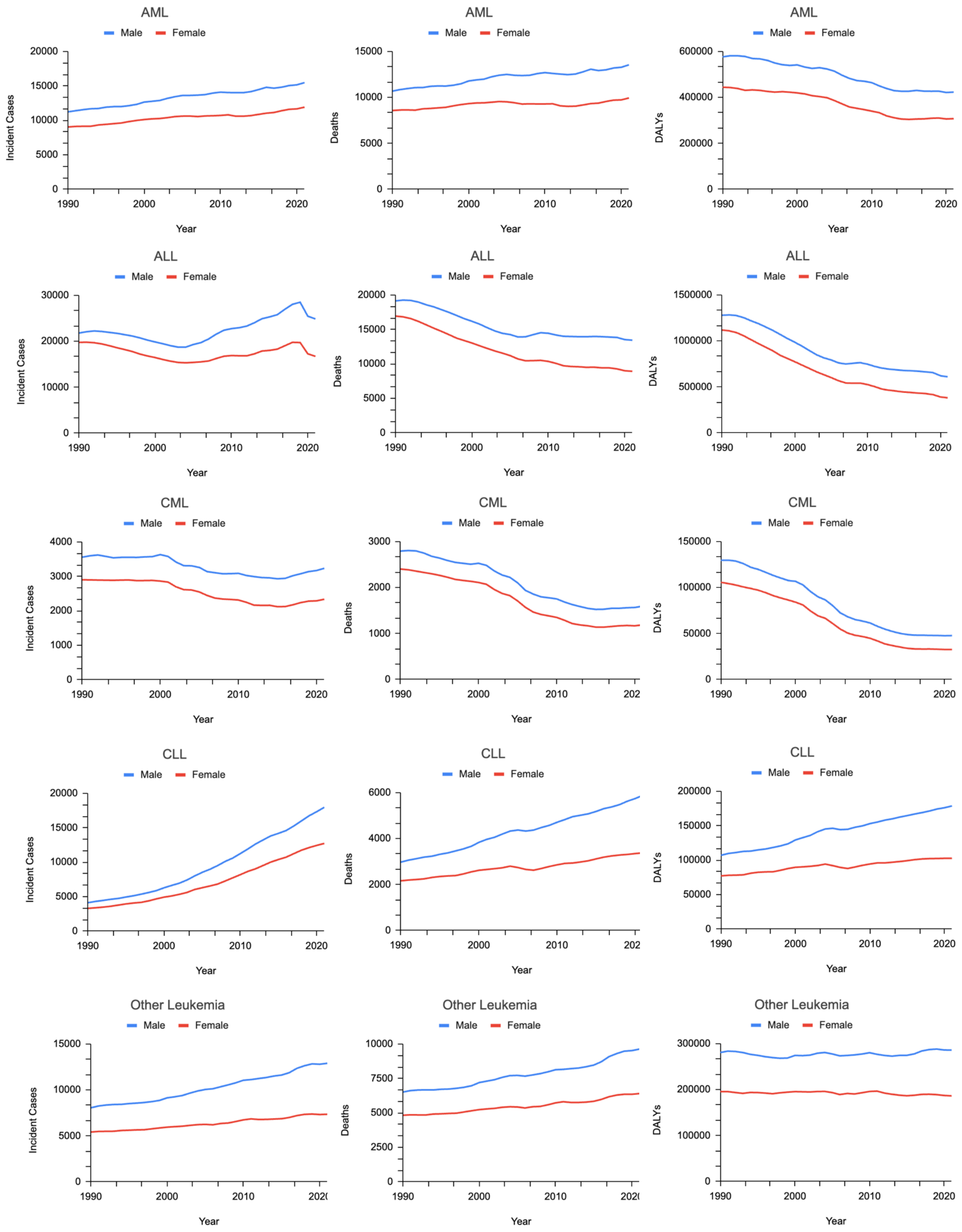

Supplementary Figure S5: China – Hematological Malignancy Burden by Age, 1990 and 2021.

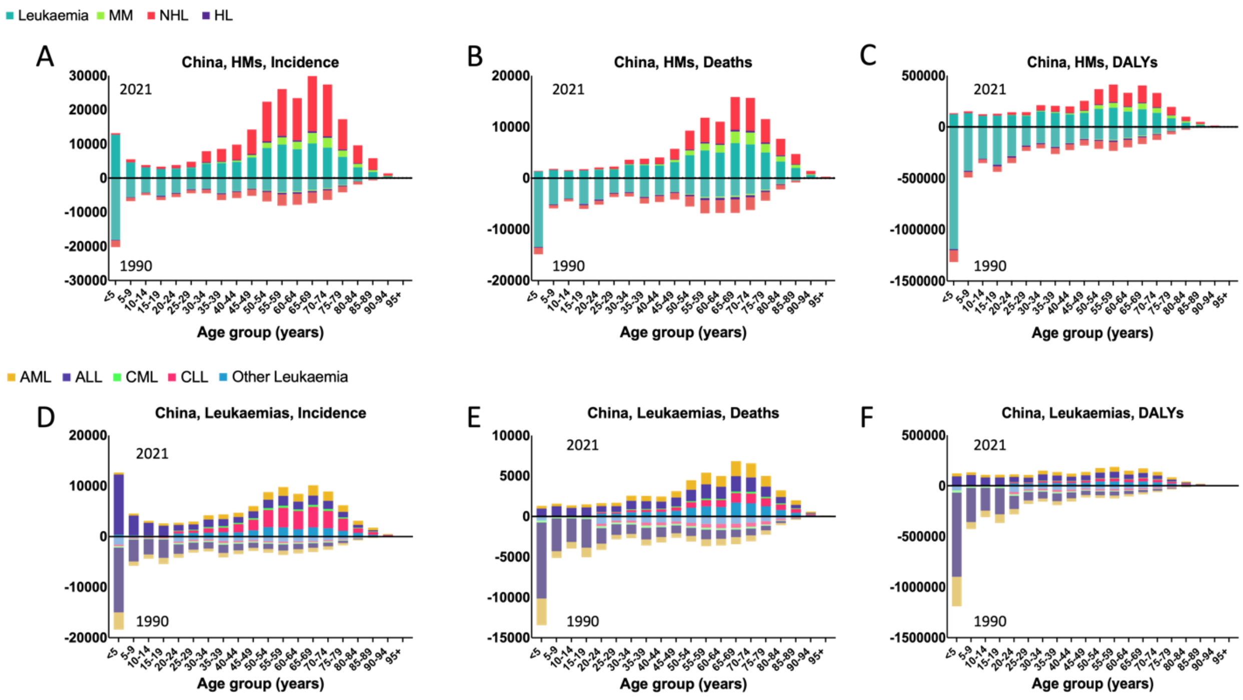

Supplementary Figure S6: China – Trends in Leukemia Burden from 1990 to 2021. (A) Incident cases, (B) Deaths, and (C) DALYs for AML, ALL, CML, CLL, Other leukemia. (D) Age-standardized incidence rate (ASIR), (E) Age-standardized mortality rate (ASMR), and (F) Age-standardized DALY rate (ASDALY) for AML, ALL, CML, CLL, Other leukemia. (G) Ratio of male to female ASIR, (H) Ratio of male to female ASMR, and (I) Ratio of male to female ASDALYR for AML, ALL, CML, CLL, Other leukemia.

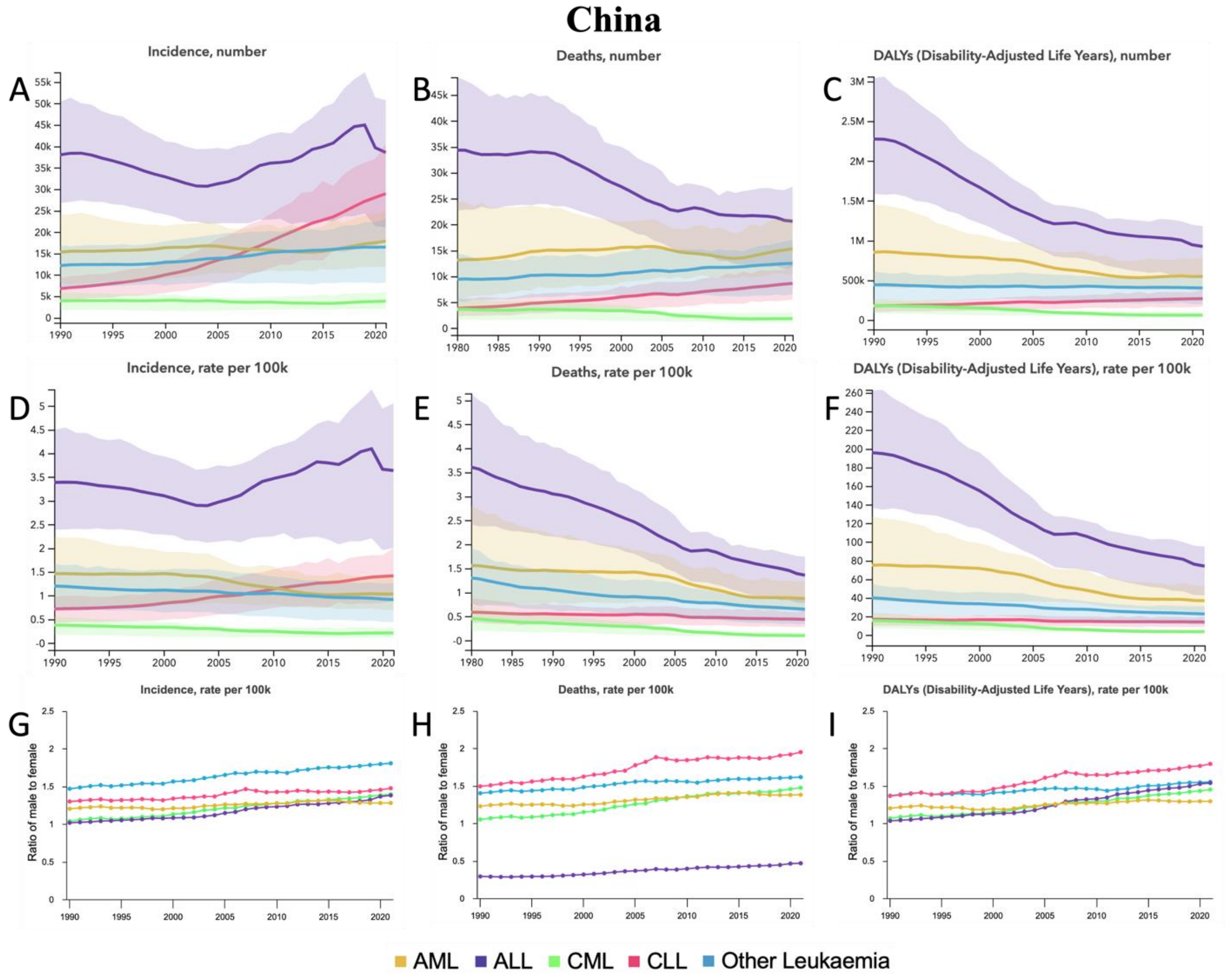

Supplementary Figure S7: Japan – Hematological Malignancy Burden by Age, 1990 and 2021.

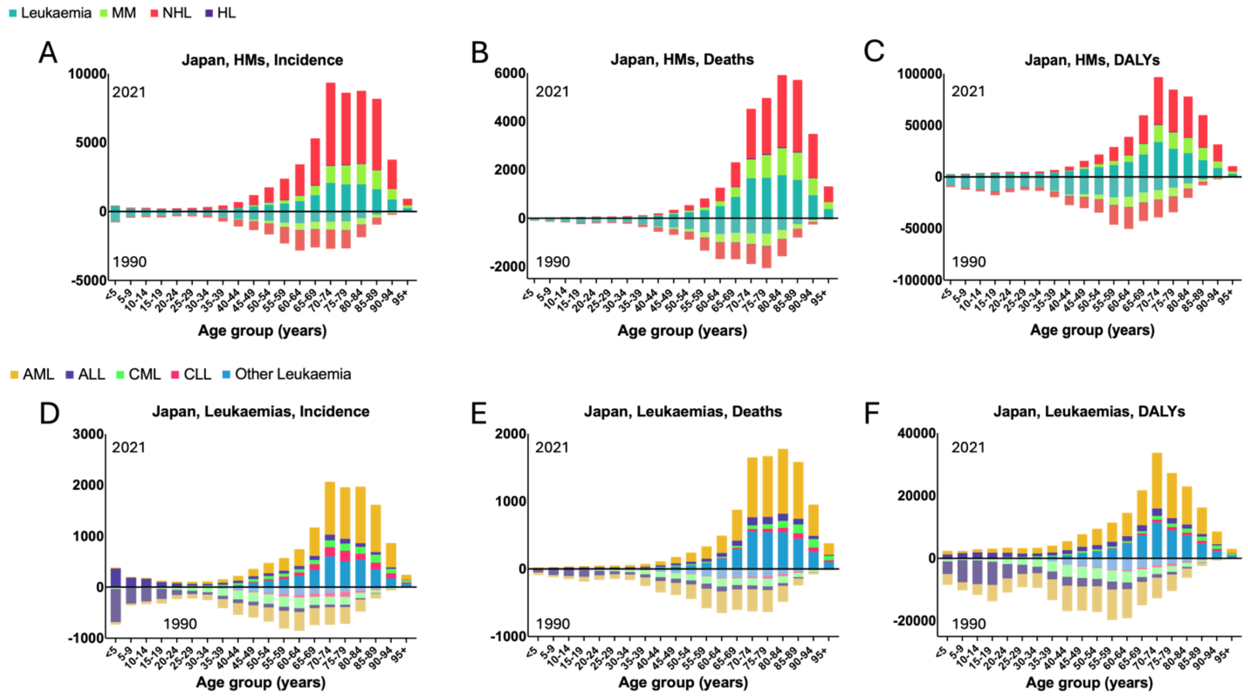

Supplementary Figure S8: Japan – Trends in Leukemia Burden from 1990 to 2021. (A) Incident cases, (B) Deaths, and (C) DALYs for AML, ALL, CML, CLL, Other leukemia. (D) Age-standardized incidence rate (ASIR), (E) Age-standardized mortality rate (ASMR), and (F) Age-standardized DALY rate (ASDALY) for AML, ALL, CML, CLL, Other leukemia. (G) Ratio of male to female ASIR, (H) Ratio of male to female ASMR, and (I) Ratio of male to female ASDALYR for AML, ALL, CML, CLL, Other leukemia.

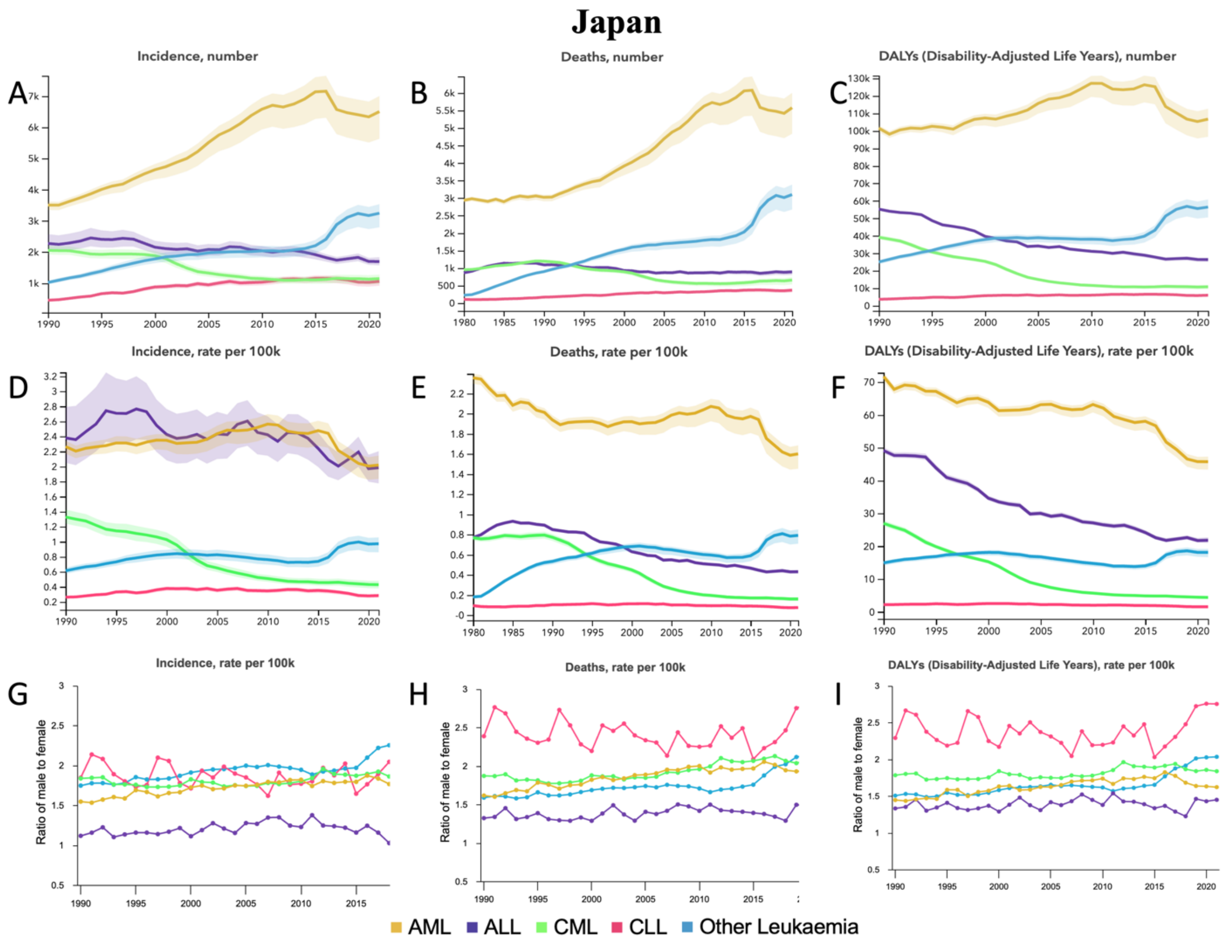

Supplementary Figure S9: North Korea – Hematological Malignancy Burden by Age, 1990 and 2021.

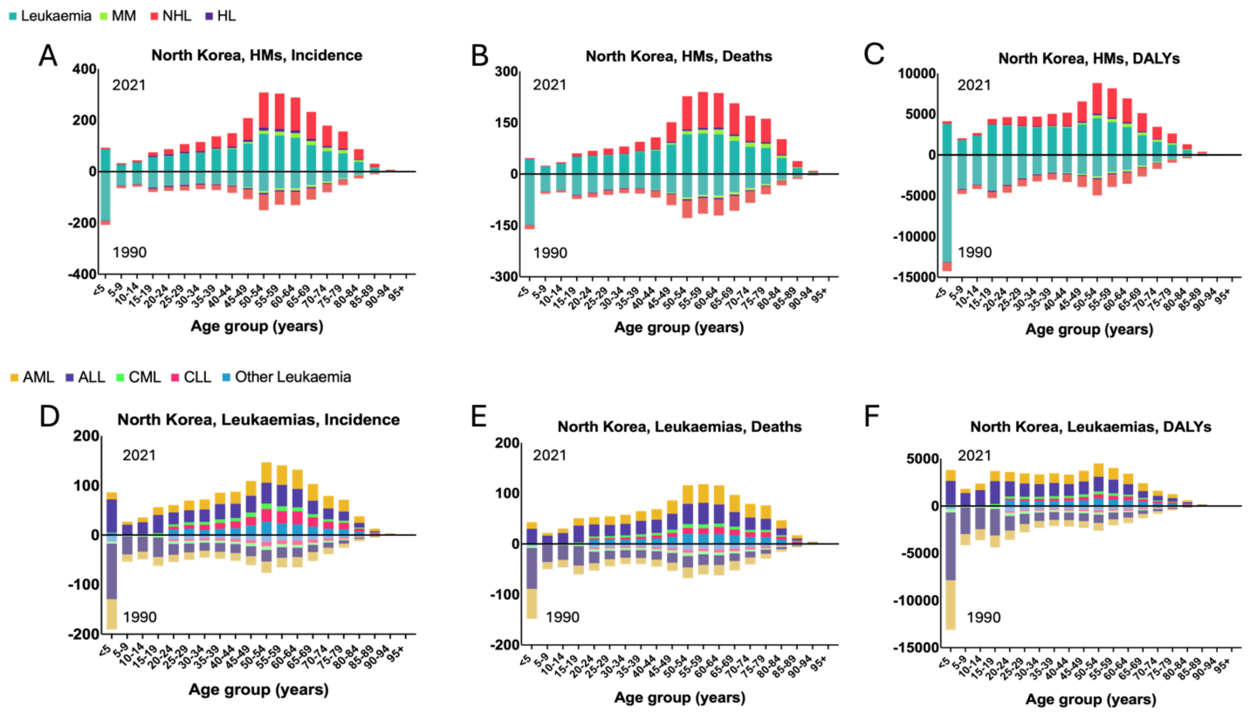

Supplementary Figure S10: North Korea – Trends in Leukemia Burden from 1990 to 2021. (A) Incident cases, (B) Deaths, and (C) DALYs for AML, ALL, CML, CLL, Other leukemia. (D) Age-standardized incidence rate (ASIR), (E) Age-standardized mortality rate (ASMR), and (F) Age-standardized DALY rate (ASDALY) for AML, ALL, CML, CLL, Other leukemia. (G) Ratio of male to female ASIR, (H) Ratio of male to female ASMR, and (I) Ratio of male to female ASDALYR for AML, ALL, CML, CLL, Other leukemia.

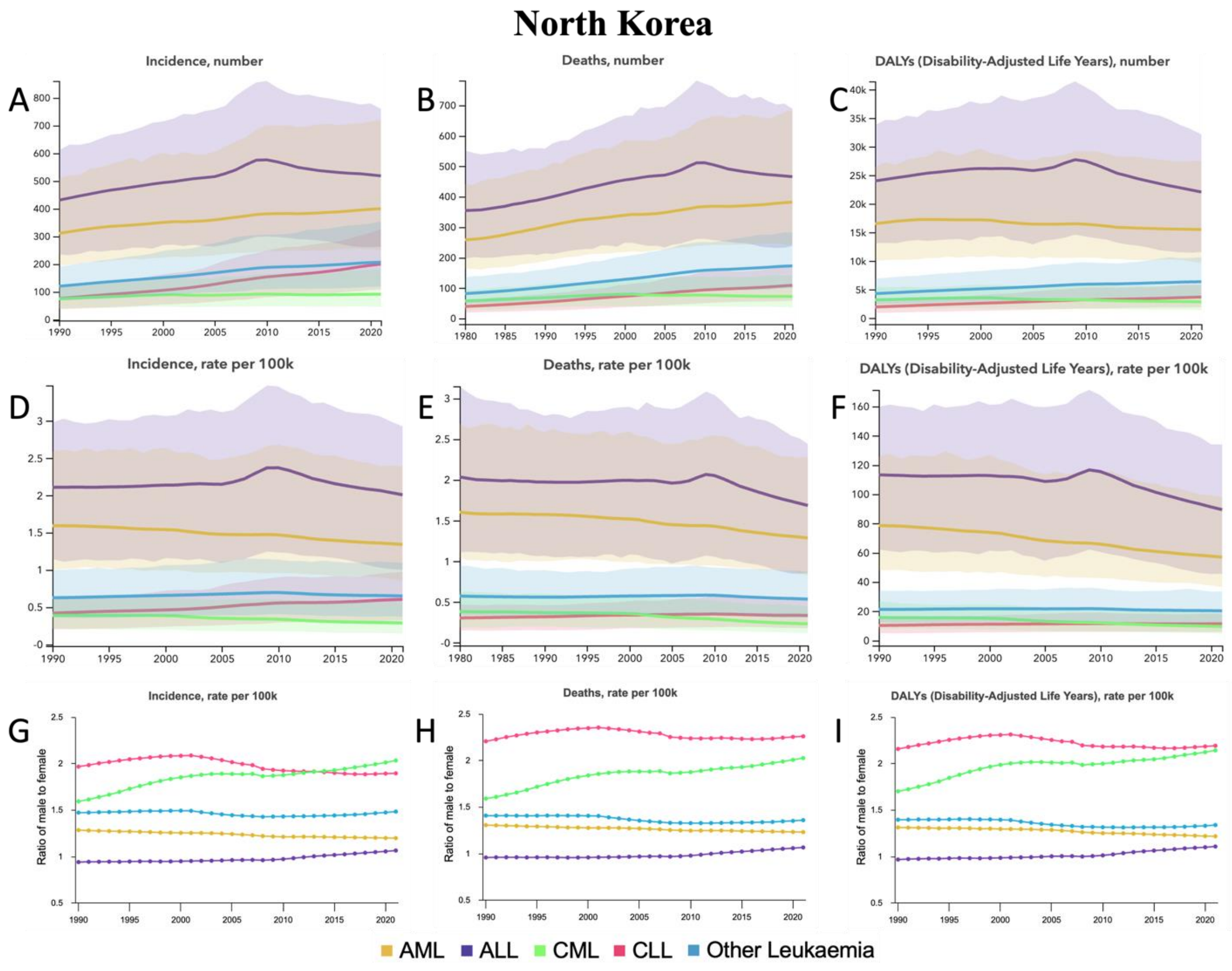

Supplementary Figure S11: South Korea – Hematological Malignancy Burden by Age, 1990 and 2021.

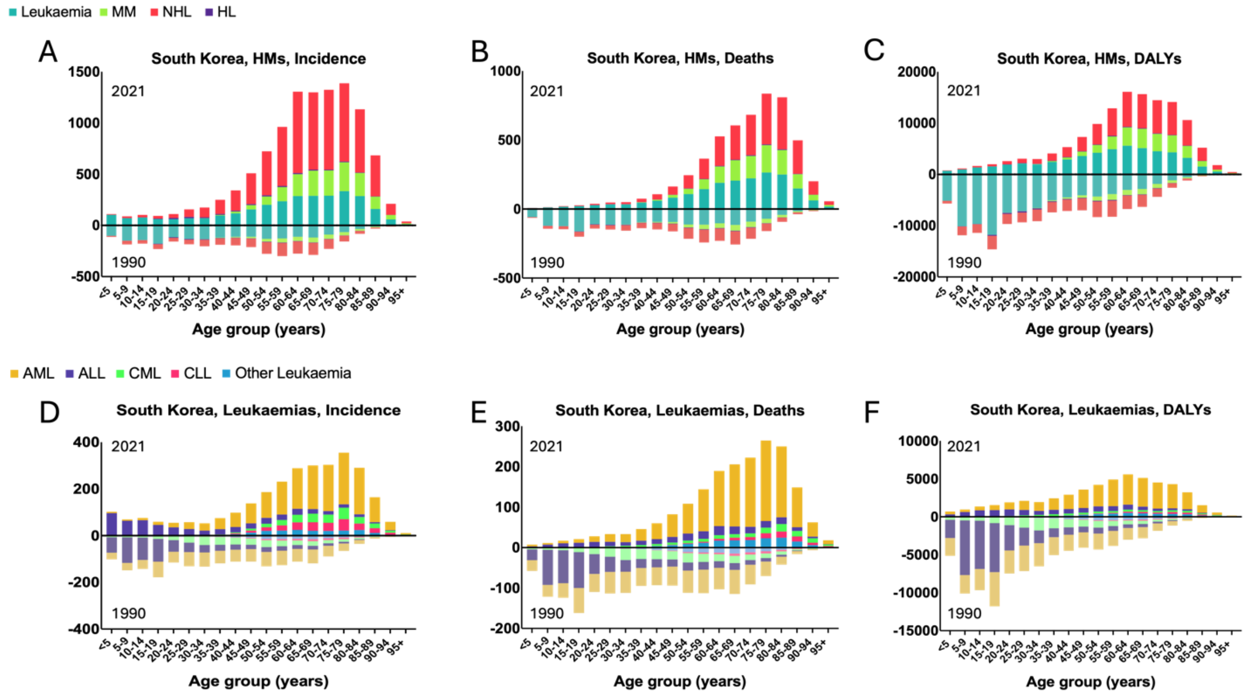

Supplementary Figure S12: South Korea – Trends in Leukemia Burden from 1990 to 2021. (A) Incident cases, (B) Deaths, and (C) DALYs for AML, ALL, CML, CLL, Other leukemia. (D) Age-standardized incidence rate (ASIR), (E) Age-standardized mortality rate (ASMR), and (F) Age-standardized DALY rate (ASDALY) for AML, ALL, CML, CLL, Other leukemia. (G) Ratio of male to female ASIR, (H) Ratio of male to female ASMR, and (I) Ratio of male to female ASDALYR for AML, ALL, CML, CLL, Other leukemia.

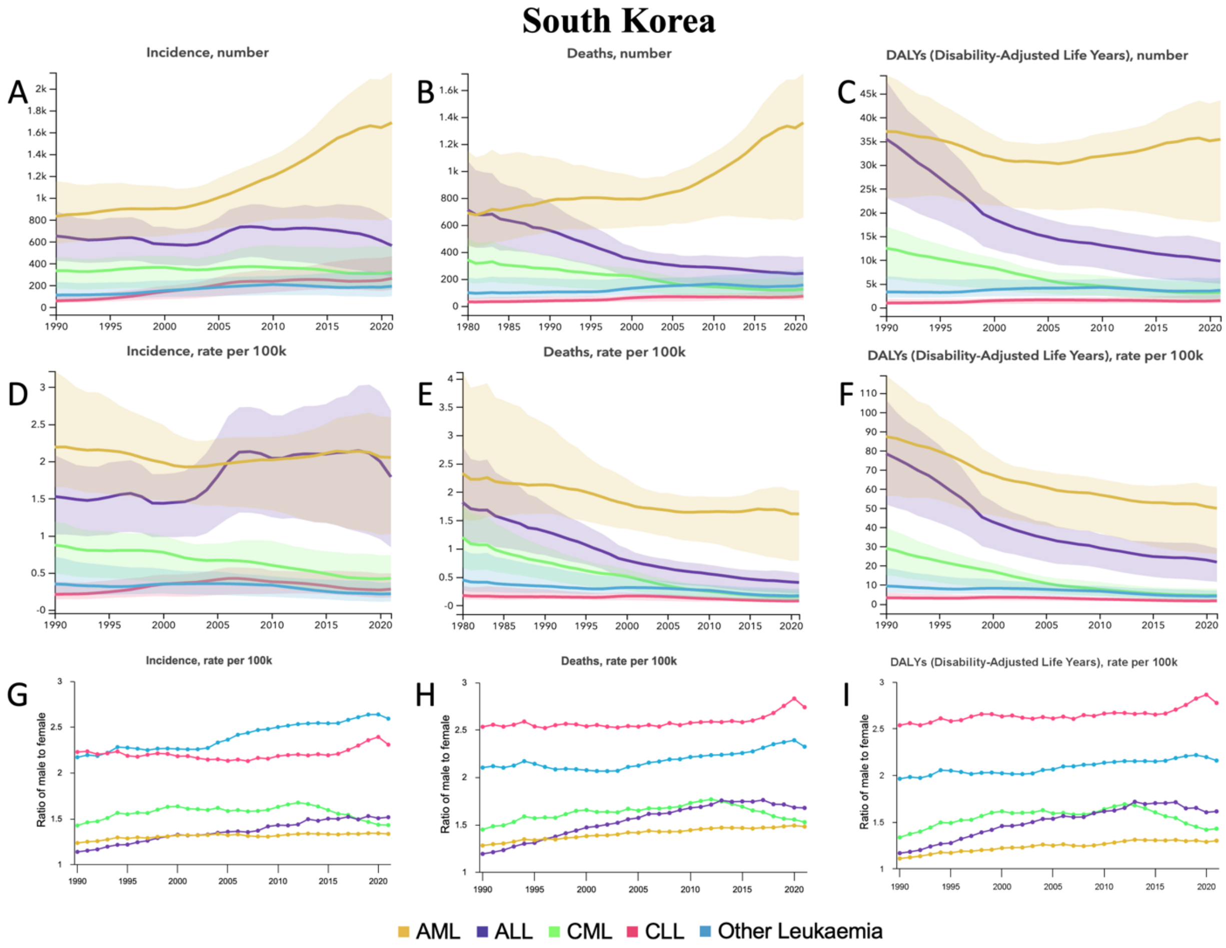

Supplementary Figure S13: Mongolia – Hematological Malignancy Burden by Age, 1990 and 2021.

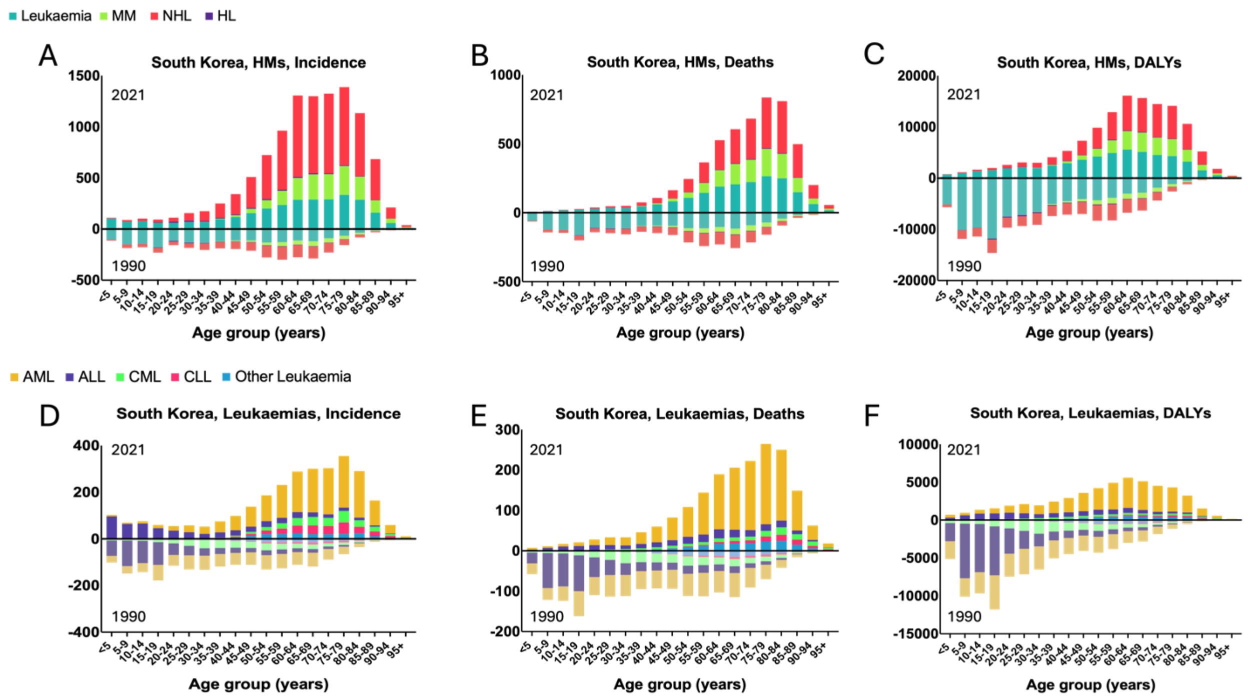

Supplementary Figure S14: Mongolia – Trends in Leukemia Burden from 1990 to 2021. (A) Incident cases, (B) Deaths, and (C) DALYs for AML, ALL, CML, CLL, Other leukemia. (D) Age-standardized incidence rate (ASIR), (E) Age-standardized mortality rate (ASMR), and (F) Age-standardized DALY rate (ASDALY) for AML, ALL, CML, CLL, Other leukemia. (G) Ratio of male to female ASIR, (H) Ratio of male to female ASMR, and (I) Ratio of male to female ASDALYR for AML, ALL, CML, CLL, Other leukemia.

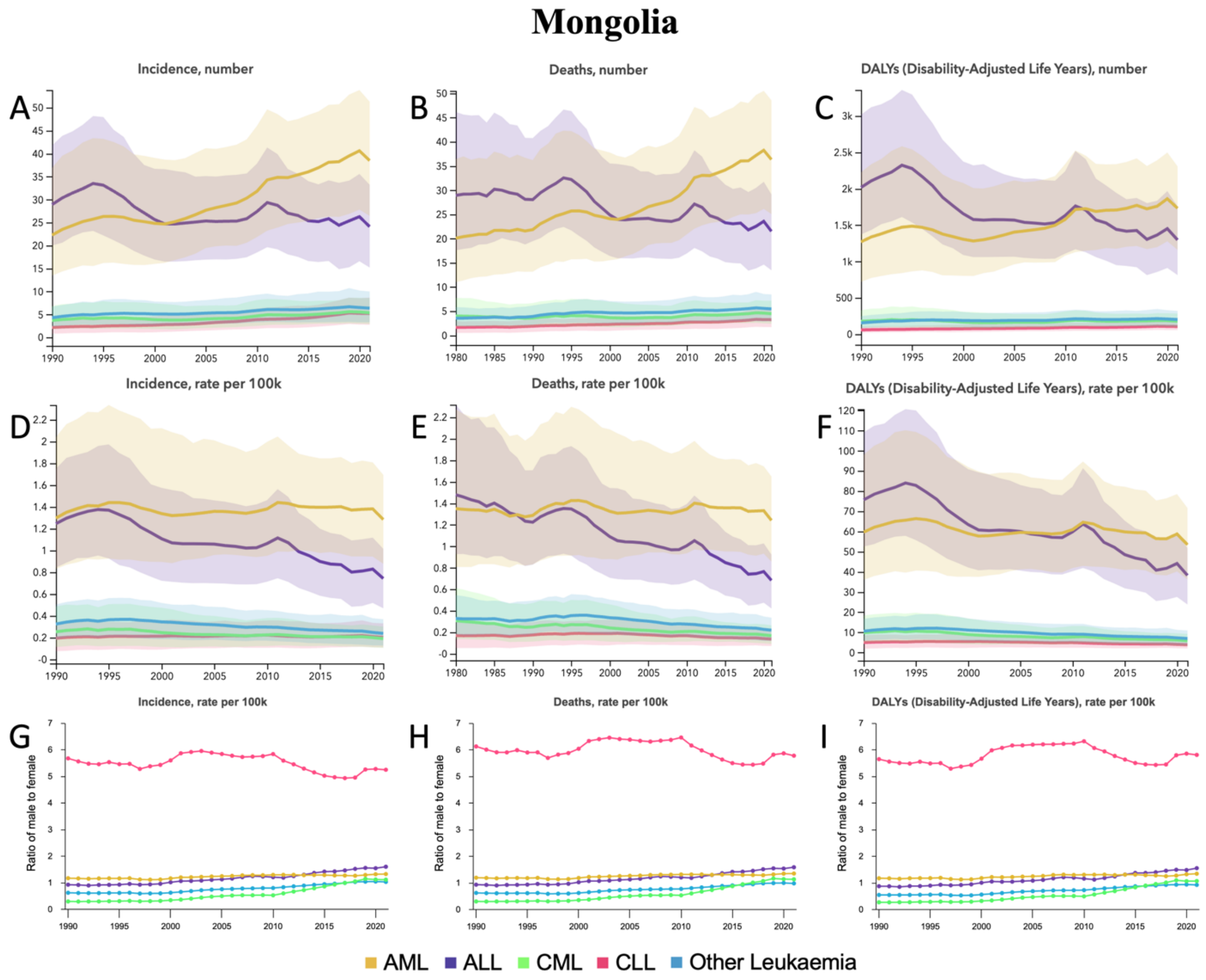

Supplementary Figure S15: Taiwan – Hematological Malignancy Burden by Age, 1990 and 2021.

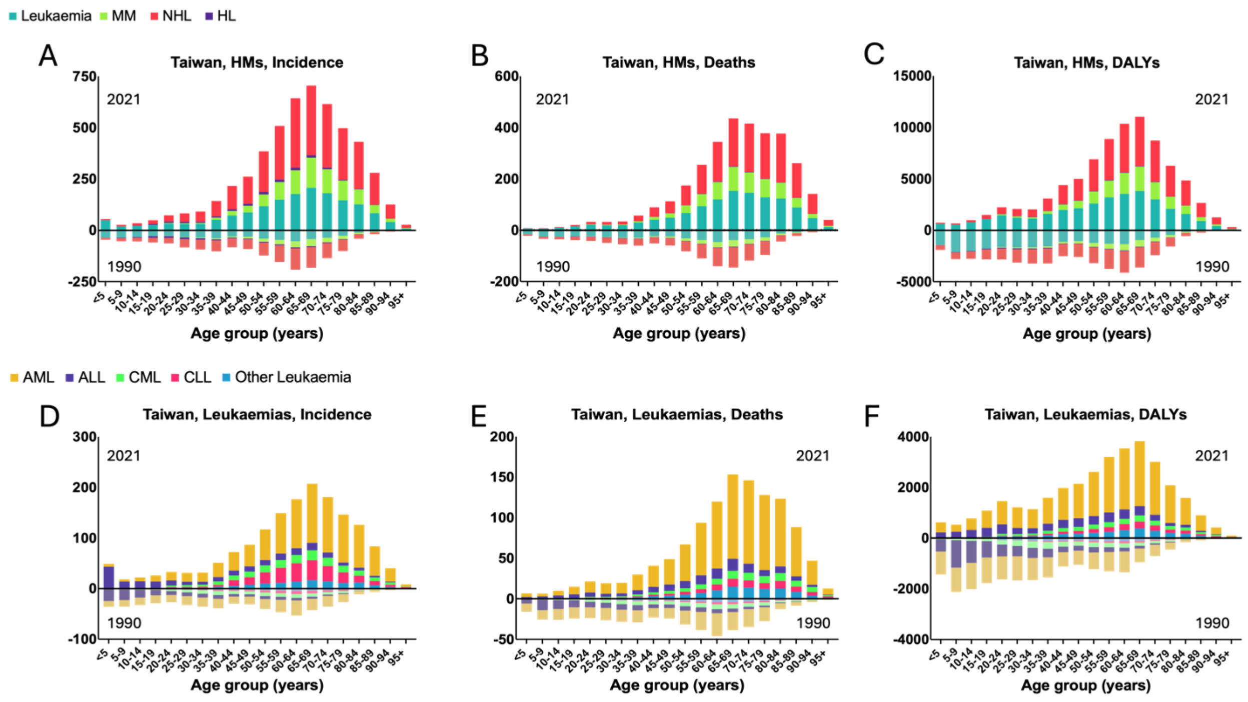

Supplementary Figure S16: Taiwan – Trends in Leukemia Burden from 1990 to 2021. (A) Incident cases, (B) Deaths, and (C) DALYs for AML, ALL, CML, CLL, Other leukemia. (D) Age-standardized incidence rate (ASIR), (E) Age-standardized mortality rate (ASMR), and (F) Age-standardized DALY rate (ASDALY) for AML, ALL, CML, CLL, Other leukemia. (G) Ratio of male to female ASIR, (H) Ratio of male to female ASMR, and (I) Ratio of male to female ASDALYR for AML, ALL, CML, CLL, Other leukemia.

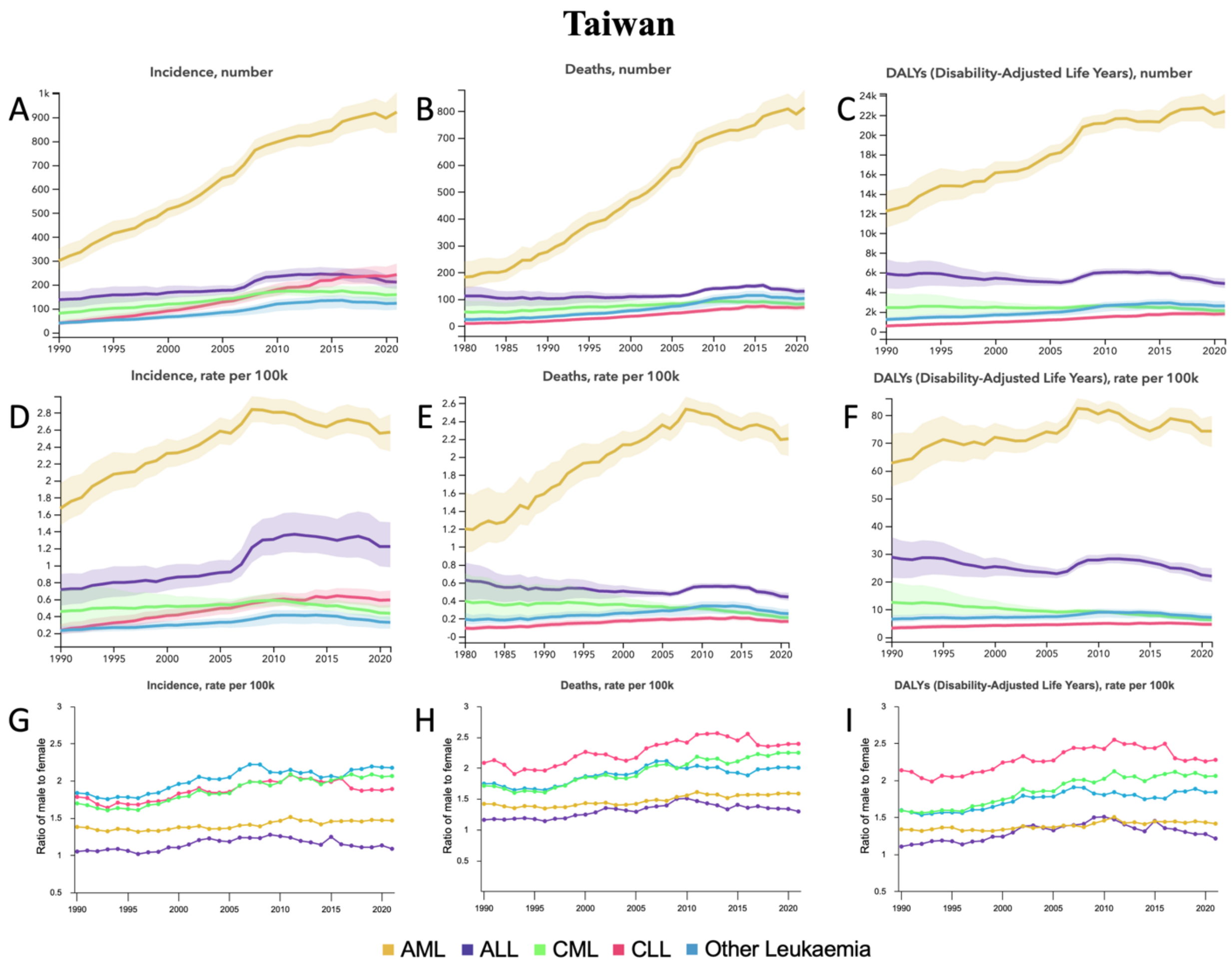

Supplement: Supplementary file 1 [file jcm-14-08381-s001.zip › jcm-3948760-supplementary.pdf]
